# A novel and conserved protein AHO-3 is required for thermotactic plasticity associated with feeding states in *Caenorhabditis elegans*

**DOI:** 10.1111/j.1365-2443.2012.01594.x

**Published:** 2012-05

**Authors:** Nana Nishio, Akiko Mohri-Shiomi, Yukuo Nishida, Naoya Hiramatsu, Eiji Kodama-Namba, Kotaro D Kimura, Atsushi Kuhara, Ikue Mori

**Affiliations:** 1 Group of Molecular Neurobiology, Division of Biological Science, Graduate School of Science, Nagoya UniversityFurou-cho, Nagoya 464-8602, Japan; 2 Core Research of Evolutional Science & Technology AgencyJapan

## Abstract

Although a large proportion of molecules expressed in the nervous system are conserved from invertebrate to vertebrate, functional properties of such molecules are less characterized. Here, we show that highly conserved hydrolase AHO-3 acts as a novel regulator of starvation-induced thermotactic plasticity in *Caenorhabditis elegans*. As wild-type animals, *aho-3* mutants migrated to the cultivation temperature on a linear thermal gradient after cultivation at a particular temperature with food. Whereas wild-type animals cultivated under food-deprived condition showed dispersed distribution on the gradient, *aho-3* mutants exhibited tendency to migrate toward higher temperature. Such an abnormal behavior was completely rescued by the expression of human homologue of AHO-3, indicating that the molecular function of AHO-3 is highly conserved between nematode and human. The behavioral regulation by AHO-3 requires the N-terminal cysteine cluster, which ensures the proper subcellular localization of AHO-3 to sensory endings. Double-mutant analysis suggested that AHO-3 acts in the same pathway with ODR-3, a heterotrimeric G protein alpha subunit. Our results unveiled a novel neural protein in *C. elegans*, confirming its conserved role in behavioral regulation.

## Introduction

The nervous system is one of the most distinctive organs in the animal kingdom. By virtue of that, animals can sense environmental stimuli, memorize the information and modify their behavior. A large fraction of proteins expressed in the nervous system are presumed to be conserved throughout the animal kingdom (Hunt-Newbury *et al.* 2007; Von Stetina *et al.* 2007) (http://www.ebi.ac.uk/gxa/). Comparative analyses elucidated that at least 38% of the 20 250 total *Caenorhabditis elegans* genes share homology with human genes (Lai *et al.* 2000; Shaye & Greenwald 2011) and that more than half of the 2500 *C. elegans* transcripts expressed in the nervous system have mammalian homologues (Von Stetina *et al.* 2007). Characterizations of the conserved neural components through animal species have provided important general insights into the mechanisms of the neural function. For example, G protein signaling pathway is essential for olfactory transduction (Buck 1996; Krieger & Breer 1999; Bargmann 2006) and phototransduction (Fu & Yau 2007; Wang & Montell 2007; Liu *et al.* 2010), cAMP response element–binding (CREB) protein pathway is a key mechanism for memory formation (Kandel 2001; Josselyn & Nguyen 2005; Kauffman *et al.* 2010; Nishida *et al.* 2011), and dopamine signaling is involved in reward learning and responses to food (Schwaerzel *et al.* 2003; Barron *et al.* 2010). Nevertheless, the neural functions of many other conserved molecules in the nervous systems still remain unknown.

*Caenorhabditis elegans* thermotaxis provides a behavioral plasticity paradigm, in which temperature preferences are modified by their cultivation temperature and feeding state (Hedgecock & Russell 1975; Mohri *et al.* 2005). After cultivation at a certain temperature with food, animals migrate to the cultivation temperature on a thermal gradient without food (Hedgecock & Russell 1975; Mohri *et al.* 2005; Ito *et al.* 2006). In contrast, animals cultivated without food disperse from the cultivation temperature (Hedgecock & Russell 1975; Mohri *et al.* 2005). This behavioral change has been called different names such as ‘thermotactic plasticity induced by starvation’ (Mohri *et al.* 2005), ‘temperature-food associative learning’ (Kuhara & Mori 2006) or ‘integrative behavior for temperature and feeding state’ (Kodama *et al.* 2006). We designated this behavior ‘thermotactic plasticity’ in this article.

To investigate the molecular and neural mechanisms underlying thermotactic plasticity, we previously performed a genetic screen to isolate the mutants defective in thermotactic plasticity, which were designated *aho* (*a*bnormal *h*unger *o*rientation) mutants (Mohri *et al.* 2005). Of these, *aho-2(nj32)* mutants migrated to the cultivation temperature in both well-fed and starved conditions (Mohri *et al.* 2005; Kodama *et al.* 2006). We found that *aho-2(nj32)* mutants exhibited a deletion in the *ins-1* gene encoding insulin homologue and showed that insulin-like signaling pathway modulates the neuronal activity of interneurons required for the execution of thermotaxis (Kodama *et al.* 2006).

In this study, we identified and analyzed the gene responsible for the *aho-3(nj15)* mutant that has distinct abnormality in thermotactic plasticity. Whereas well-fed *aho-3(nj15)* mutants migrated to the cultivation temperature on a temperature gradient, starved *aho-3(nj15)* mutants showed tendency to migrate toward higher temperature. This abnormal phenotype is different from that of *aho-2(nj32)* mutants. We showed that the *aho-3* gene encodes a novel and highly conserved hydrolase. The abnormality in thermotactic plasticity of *aho-3* mutants was completely rescued by expressing human homologue of AHO-3, FAM108B1 protein, indicating that the molecular property is highly conserved between nematode and human. It was previously reported that rodent homologues of AHO-3, FAM108 proteins, are found in the membrane fraction of the brain proteome (Blankman *et al.* 2007; Kang *et al.* 2008). In addition, other study showed that the conserved N-terminal cysteine cluster of human FAM108 proteins is necessary for its plasma membrane localization (Martin & Cravatt 2009). We show here that AHO-3 acts in sensory neurons and localizes to sensory endings. Furthermore, the N-terminal cysteine cluster of AHO-3 is necessary for its subcellular localization and for its function in thermotactic plasticity. Double-mutant analysis suggested that AHO-3 acts in the same pathway with ODR-3, a heterotrimeric G protein alpha subunit, which is localized to sensory endings (Roayaie *et al.* 1998; Bargmann 2006). Our results suggest that evolutionarily conserved AHO-3 has important functions in the nervous system for behavioral plasticity.

## Results

### *aho-3* mutants show abnormality in thermotactic plasticity associated with feeding states

**Figure 1:**
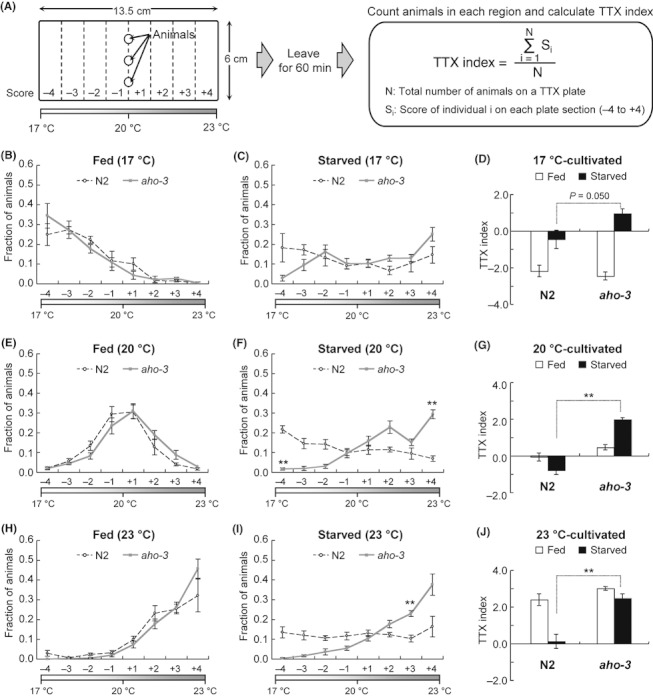
*aho-3* mutants show abnormalities in thermotactic plasticity. (A) Procedures for population thermotaxis assay using a linear thermal gradient (Ito *et al.* 2006); 40–300 animals cultivated at 17, 20 or 23 °C were placed on the centerline of a TTX (thermotaxis) plate and allowed to move freely for 60 min. The animals in each region (from −4 to +4) were counted, and the TTX index was calculated as described. A 17–23 °C thermal gradient was always used in this study, except for Figure S1 in Supporting Information (see Experimental procedures for detail). (B–J) Thermotaxis of well-fed or starved wild-type N2 animals and *aho-3(nj15)* mutants that were cultivated at 17 °C (B–D), 20 °C (E–G) or 23 °C (H–J). *n* ≥ 4 assays. Error bars represent SEM. In (B, C, E, F and H, I), statistical significance of values in each region was tested by unpaired *t*-test with the Dunn–Sidak correction for multiple comparisons; *P < 0.05; **P < 0.01. In (D, G and J), statistical significance of TTX indices was tested by unpaired *t*-test in comparisons of well-fed N2 animals vs well-fed *aho-3* mutants or starved N2 animals vs starved *aho-3* mutants; *P < 0.05; **P < 0.01.

We have previously reported that *C. elegans* exhibits thermotactic plasticity depending on their feeding states using the individual thermotaxis assay with a nonlinear thermal gradient; most of well-fed wild-type animals migrate to their cultivation temperature, whereas few starved animals migrate to their cultivation temperature (Mohri *et al.* 2005). In this study, we performed the population thermotaxis assay with a linear thermal gradient, which is suitable for quantitatively assessing the migration ability toward a certain temperature (Ito *et al.* 2006). We used the linear thermal gradient ranging from 17 to 23 °C with 20 °C at the center (Fig. 1A). After cultivation at 17, 20 or 23 °C under well-fed condition, wild-type N2 animals migrated to their cultivation temperature (Fig. 1B,E,H). By contrast, after cultivation at 17, 20 or 23 °C under food-deprived (starved) condition for 3, 2 or 1 h, respectively, most of the starved wild-type animals dispersed and did not migrate to their cultivation temperature (Fig. 1C,F,I; see ‘Experimental procedures’ for details on the starvation conditioning).

To investigate the molecular components in thermotactic plasticity, we conducted a forward genetic screen and isolated *aho-3(nj15)* mutants that display abnormality in thermotactic plasticity (Mohri *et al.* 2005). Similar to the well-fed wild-type animals, most of the well-fed *aho-3(nj15)* mutants cultivated at 17, 20 or 23 °C migrated to their cultivation temperature (Fig. 1B,D,E,G,H,J; *P* > 0.05; in unpaired *t-*test compared TTX indices), although distributions of *aho-3(nj15)* animals seemed to slightly shift toward higher temperature than those of wild-type animals after cultivation at 20 and 23 °C.

Starved *aho-3(nj15)* mutants conditioned at 17 °C seemed to migrate slightly higher temperature than the starved wild-type animals (Fig. 1C,D), but it was not statistically significant in unpaired *t-*test (*P* = 0.050; compared TTX indices). Unlike starved wild-type animals, starved *aho-3(nj15)* mutants conditioned at 20 °C migrated to the higher-temperature regions (+2, +3 and +4) than their cultivation temperature (Fig. 1F,G; *P* < 0.01; compared TTX indices), and starved *aho-3(nj15)* mutants conditioned at 23 °C also migrated to the high-temperature regions (+3 and +4) (Fig. 1I,J; *P* < 0.01; compared TTX indices). These results indicate that starved *aho-3(nj15)* mutants show abnormal thermophilic phenotype after 20 or 23 °C cultivation; they migrate to the high-temperature regions on thermal gradient ranging from 17 to 23 °C.

There are two possible explanations for the defect of *aho-3(nj15)* mutants: starved *aho-3(nj15)* mutants always seek out 23 °C regardless of their cultivation temperature or just higher temperatures. To examine these possibilities, we used a linear thermal gradient ranging from 20 to 26 °C with 23 °C at the center (Fig. S1A–I in Supporting Information). The starved wild-type animals conditioned at 17 or 23 °C dispersed (Fig. S1B,H in Supporting Information), whereas the starved wild-type animals conditioned at 20 °C migrated to lower-temperature region (Fig. S1E in Supporting Information). *aho-3(nj15)* mutants also showed abnormality in thermotactic plasticity on this thermal gradient. Few starved *aho-3(nj15)* mutants conditioned at 17 or 23 °C migrated to lower-temperature region (Fig. S1B,H in Supporting Information), and starved *aho-3(nj15)* mutants conditioned at 20 °C showed the weaker cryophilic phenotype than wild-type animals (Fig. S1E in Supporting Information). The TTX indices of starved *aho-3* mutants were significantly higher than those of starved wild-type animals (Fig. S1C,F,I in Supporting Information). These results suggest that *aho-3(nj15)* mutants do not always seek out 23 °C but rather show a tendency to accumulate higher temperature than wild-type animals after starvation.

To investigate whether *aho-3(nj15)* mutants have abnormalities in other behavioral modification, we performed a salt chemotaxis learning assay (Saeki *et al.* 2001; Tomioka *et al.* 2006) and an integration test for two opposite chemosensory stimuli, repellent Cu^2+^ ion and attractant diacetyl (Ishihara *et al.* 2002). *aho-3(nj15)* mutants showed defects in both the salt learning behavior and integration behavior (Fig. S2A–F in Supporting Information), whereas they show normal chemotaxis to salt and diacetyl and avoidance of Cu^2+^ ion (Mohri *et al.* 2005) (Fig. S2G–H in Supporting Information). Altogether, these results suggest that *aho-3(nj15)* mutants have abnormalities not only in thermotactic plasticity but also in multiple complex behaviors.

### The *aho-3* gene encodes a novel and highly conserved hydrolase

**Figure 2:**
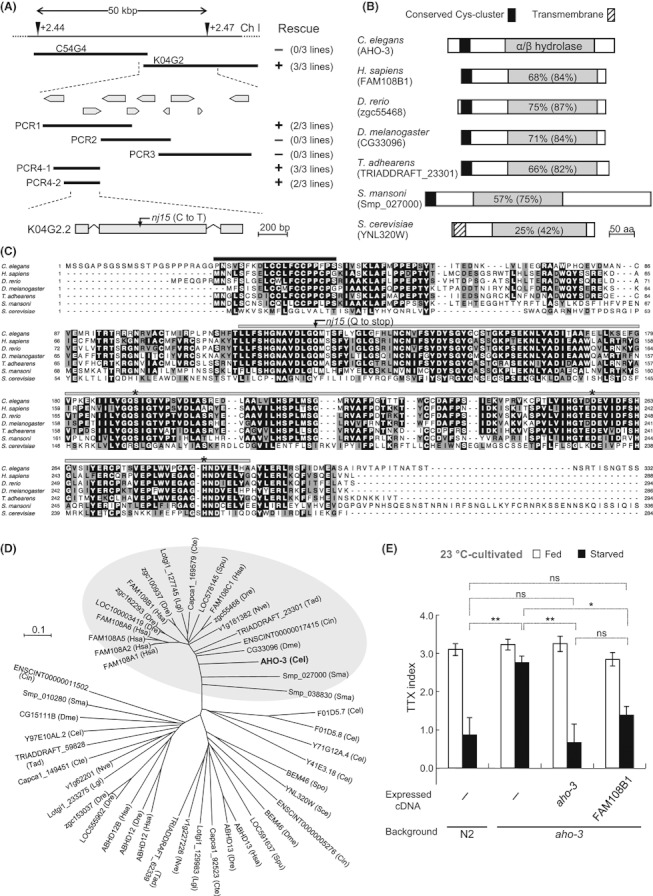
The *aho-3* gene encodes a novel protein, which highly conserved among animal species. (A) Position of the *aho-3* gene on chromosome I. Arrowheads show the locations of single-nucleotide polymorphisms (SNPs). Results of rescue experiments for abnormal thermotactic plasticity of *aho-3(nj15)* mutants are indicated as + (rescued) or − (not rescued) on the right side; the rescue experiments were performed with individual thermotaxis assay. Numbers in parentheses indicate the fraction of rescued lines. The gene structure for K04G2.2 is depicted (bottom). The *nj15* mutation is shown. (B) Predicted structure of AHO-3 homologues and similar protein. Percentages indicate amino acid identity and similarity (given in parentheses) in the alpha/beta-hydrolase domain between *Caenorhabditis elegans* AHO-3 and other proteins. (C) Alignment of AHO-3 homologues and similar protein; these proteins are the same as (B). Black bar represents cysteine cluster motif, and gray bar represents alpha/beta-hydrolase domain. Asterisks represent predicted catalytic residues. The *nj15* mutation results in Q to STOP at position 127. The C-terminal sequence of *Schistosoma mansoni* protein is omitted here. (D) Unrooted dendrogram of AHO-3 homologues and similar proteins in 11 animals and two yeasts. Gray ellipse shows AHO-3 homologue group, in which proteins share >70% amino acid sequence similarity with *C. elegans* AHO-3 in the alpha/beta-hydrolase domains and have the N-terminal cysteine cluster. Cel, *Caenorhabditis elegans*; Hsa, *Homo sapiens*; Dre, *Danio rerio*; Cin, *Ciona intestinalis*; Spu, *Strongylocentrotus purpuratus*; Dme, *Drosophila melanogaster*; Cte, *Capitella teleta*; Lgi, *Lottia gigantea*; Sma, *Schistosoma mansoni*; Nve, *Nematostella vectensis*; Tad, *Trichoplax adhaerens*; Sce, *Saccharomyces cerevisiae*; Spo, *Schizosaccharomyces pombe*. (E) Rescue experiment for the abnormality of *aho-3(nj15)* mutants with human AHO-3 homologue. Thermotaxis of control animals and transgenic *aho-3(nj15)* mutants expressing *C. elegans* AHO-3 or human FAM108B1 pan-neuronally. Test animals were cultivated at 23 °C. *n* ≥ 4 assays. Error bars represent SEM. Tukey’s test was used for multiple comparisons among TTX indices of starved animals; *P < 0.05; **P < 0.01; ns, not significant (*P* > 0.05).

To identify the gene responsible for the abnormal thermotactic plasticity of *aho-3(nj15)* mutants, we performed genetic mapping with the snip-single-nucleotide polymorphisms (SNPs) method (Wicks *et al.* 2001), and rescue experiments with cosmids or PCR fragments. The *aho-3(nj15)* mutation was mapped to a 50-kbp region on the chromosome I (Fig. 2A). We found that the abnormal thermotactic plasticity of *aho-3(nj15)* mutants was rescued by the introduction of the K04G2 cosmid and PCR fragments containing the K04G2.2 gene region (Fig. 2A and Figs S3A–D and S4A–D in Supporting Information). Through DNA sequencing, we identified a C-to-T substitution in the K04G2.2 gene of *aho-3(nj15)* animals, causing a non-sense mutation (Fig. 2A). The mutation truncated in K04G2.2 product lacking catalytic domain and thus presumably causes in a strong loss of K04G2.2 function. These results suggest that K04G2.2 is the gene responsible for *aho-3(nj15)* mutants.

We also conducted rescue experiments for the salt learning behavior and integration behavior. The defect of *aho-3(nj15)* mutants in salt chemotactic plasticity was rescued by the PCR fragment containing the K04G2.2 gene (Fig. S2B in Supporting Information), suggesting that K04G2.2 is the gene responsible for the abnormal salt learning behavior of *aho-3(nj15)* mutants. However, the abnormal integration behavior was not rescued by the PCR fragment (Fig. S2D in Supporting Information). Expression of K04G2.2 cDNA under the control of the pan-neuronal promoter seemed subtly rescued the defects, although it was not significant (Fig. S2D in Supporting Information). These results imply that K04G2.2 is not a gene responsible for the abnormal integration behavior of *aho-3(nj15)* mutants. However, it is possible that rescue of this defect by K04G2.2 requires strict dose dependency. We designated K04G2.2 as *aho-3*.

The *aho-3* gene encodes a novel protein of 332 amino acid residues that possesses an alpha/beta-hydrolase domain at its C-terminus (Fig. 2B,C). blast searches showed that AHO-3 protein is highly conserved throughout animal species; AHO-3 protein shares 47%–66% amino acid sequence identity and 61%–80% amino acid sequence similarity with FAM108 proteins in *Homo sapiens* (Chordata), zgc55468 in *Danio rerio* (Chordata), CG33096 in *Drosophila melanogaster* (Arthropoda), TRIADDRAFT_23301 in *Trichoplax adhaerens* (Placozoa), Smp_027000 in *Schistosoma mansoni* (Platyhelminthes), etc. (calculated utilizing the blastp 2.2.24+ algorithm; Fig. 2B–D and Figs S5 and S6 and Table S1 in Supporting Information). Functions of these AHO-3 homologues *in vivo* have remained completely unknown, although a few molecular properties of the AHO-3 homologues have been investigated (Blankman *et al.* 2007; Kang *et al.* 2008; Martin & Cravatt 2009; Bachovchin *et al.* 2010; Marrs *et al.* 2010). Martin and Cravatt reported that the N-terminal cysteine cluster of the human FAM108 proteins is modified with a 16-carbon fatty acid palmitate (Martin & Cravatt 2009). The N-terminal cysteine cluster is conserved in *C. elegans* AHO-3 and the predicted homologues of AHO-3 in other animal species (Fig. 2B–D and Figs S5 and S6 in Supporting Information). Although we also found similar proteins to AHO-3 in nonanimal species, *Arabidopsis thaliana* (Streptophyta), *Saccharomyces cerevisiae* (Ascomycota), *Cyanidioschyzon merolae* (Rhodophyta), *Dictyostelium discoideum* (Amoebozoa), etc., the palmitoylation motif is not conserved in those proteins (Fig. 2B–D and Figs S5 and S6 in Supporting Information).

To examine whether AHO-3 is conserved functionally among animal species, we generated transgenic animals that express *C. elegans* AHO-3 cDNA or human FAM108B1 cDNA in most of the neurons of *aho-3(nj15)* mutants, and evaluated thermotactic plasticity of those transgenic animals. Both *C. elegans* AHO-3 expression and human FAM108B1 expression fully rescued the abnormality of *aho-3(nj15)* mutants (Fig. 2E), suggesting that the molecular function of AHO-3 protein is conserved between nematode and human.

### AHO-3 functions in thermotactic plasticity in sensory neurons including AWC

**Figure 3:**
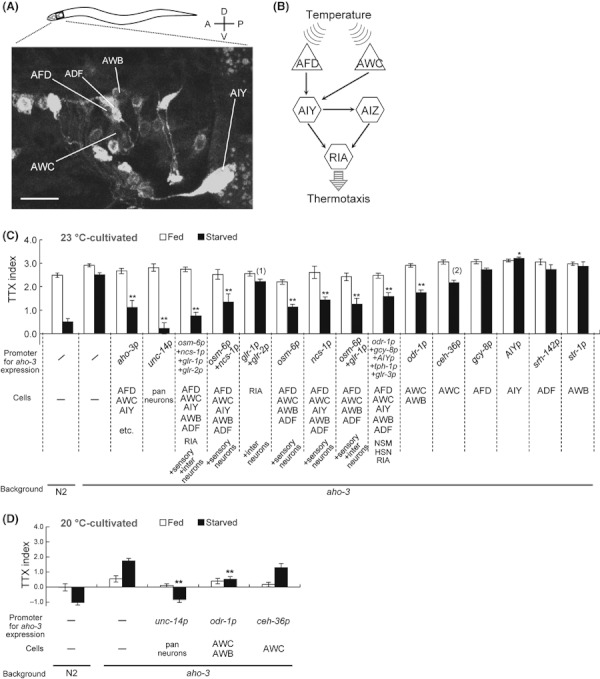
Cell-specific rescue experiments for the abnormal thermotactic plasticity of *aho-3* mutants. (A) The expression of *aho-3p::cytochrome b5::yfp* in the head of an adult wild-type animal. The general ER marker, *cytochrome b5::yfp* (Rolls *et al.* 2002), was used to show the expression in cell bodies clearer. Anterior is to the left and dorsal is up. Bars represent 10 μm. A confocal projection (z-stack = 6.4 μm) including only a part of ADF cell body is shown because the strong fluorescence in ADF in other section masks the fluorescence in the AFD cell body. (B) The simplest neural circuit model for thermotaxis (Mori & Ohshima 1995; Biron *et al.* 2008; Kuhara *et al.* 2008). AFD and AWC thermosensory neurons (triangle) and downstream AIY, AIZ and RIA interneurons (hexagons) are shown. Arrows represent synaptic connections (White *et al.* 1986). (C, D) Cell-specific rescue experiments for the abnormal thermotactic plasticity of *aho-3(nj15)* mutants. Thermotaxis of well-fed or starved animals cultivated at 23 °C (C) or at 20 °C (D). *n* ≥ 3 assays. Error bars represent SEM. Asterisks represent the comparison of starved transgenic animals with starved *aho-3* mutants by Dunnett test; *P < 0.05; **P < 0.01. (1) and (2) represent the comparison of starved transgenic animals with starved *aho-3* mutants by unpaired *t*-test; (1), *P* = 0.059; (2), *P* = 0.030.

In order to identify the cells in which AHO-3 functions, we analyzed the expression pattern of *aho-3*. We introduced into wild-type animals fluorescent reporter genes driven by *aho-3* promoter. Fluorescence was observed in several tissues (Fig. S7A,B in Supporting Information) and a subset of sensory and interneurons, including AFD (weak fluorescence), AWC (weak) and AIY (strong) neurons that are required for thermotaxis (Mori & Ohshima 1995; Biron *et al.* 2008; Kuhara *et al.* 2008), AWB neuron (weak) mediating the avoidance of repellent odors (Troemel *et al.* 1997) and serotonergic neurons HSN, ADF (strong) and NSM (weak) (Rand & Nonet 1997) (Fig. 3A,B and Fig. S7A–R in Supporting Information).

We conducted cell-specific rescue experiments for thermotactic plasticity of *aho-3(nj15)* mutants cultivated at 23 °C with a linear thermal gradient from 17 to 23 °C. Expression of *aho-3* cDNA under the control of its own promoter or pan-neuronal promoter completely rescued the abnormal thermotactic plasticity of *aho-3(nj15)* mutants (Fig. 3C). Similarly, AHO-3 expression in ∼40 pairs of sensory and interneurons also completely rescued the abnormality (Fig. 3C and Table S2 in Supporting Information). The abnormality was partially rescued by expressing AHO-3 in ∼20 pairs of sensory neurons, but not rescued by expressing in ∼20 pairs of interneurons (Fig. 3C). AHO-3 expression only in AWC and AWB sensory neurons driven by *odr-1* promoter partially rescued the abnormality, but expression in AFD, AIY, ADF or AWB neurons did not (Fig. 3C). Furthermore, the rescue efficiency of the transgenic animals expressing AHO-3 driven by *odr-1* promoter was not increased by the additional expression of AHO-3 in AFD, AIY and ADF neurons (Fig. 3C). The AHO-3 expression under the control of the *ceh-36prom3*, an AWC-specific promoter, also slightly rescued the abnormality of *aho-3(nj15)* (Fig. 3C). Altogether, these results suggest that AHO-3 functions in a subset of sensory neurons including AWC for thermotactic plasticity.

We also conducted cell-specific rescue experiments for thermotactic plasticity of *aho-3(nj15)* mutants cultivated at 20 °C using the pan-neuronal promoter, *odr-1* promoter and *ceh-36prom3* promoter (Fig. 3D). The abnormal thermotactic plasticity was rescued by the expression of AHO-3 under the pan-neuronal promoter (Fig. 3D and Fig. S8A in Supporting Information). The abnormality was partially rescued by the expression of AHO-3 in AWC and AWB under *odr-1* promoter (Fig. 3D and Fig. S8B in Supporting Information), but not significantly rescued by the expression only in AWC under *ceh-36prom3* promoter (Fig. 3D and Fig. S8C in Supporting Information). Although the site of AHO-3 function might be different depending on the cultivation temperature, it is possible that AWC-specific expression from the extrachromosome array is not stable, resulting in these different results.

**Figure 4:**
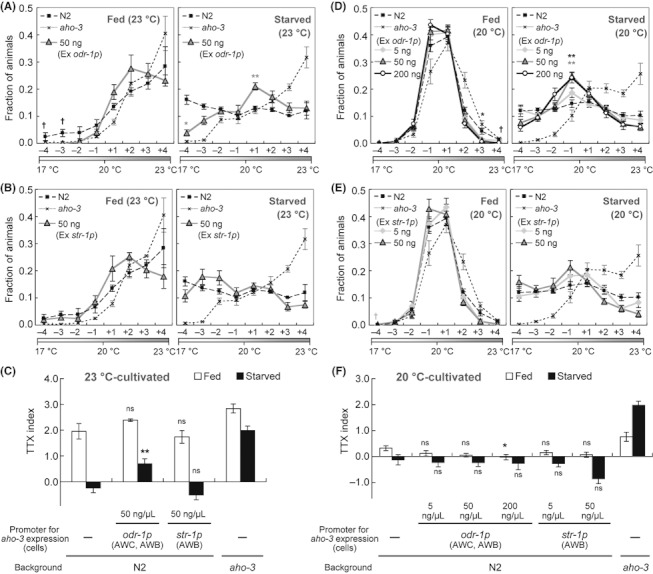
Thermotactic plasticity of animals over-expressing AHO-3. (A–F) Thermotaxis of wild-type animals, transgenic animals and *aho-3(nj15)* mutants cultivated at 23 °C (A–C) or at 20 °C (D–F) with or without food. The transgenic animals expressing excess AHO-3 in AWC and AWB under the *odr-1* promoter (A, D) or only in AWB under the *str-1* promoter (B, E) were used. *n* ≥ 3 assays. Error bars represent SEM. In (A, B and D, E), asterisks represent the comparison of values in individual eight regions by unpaired *t*-test with the Dunn–Sidak correction for multiple comparison; *P < 0.05; **P < 0.01; colors of asterisks, gray and black, represent the comparisons of N2 animals to each transgenic animals with 50 ng/μL or 200 ng/μL of *aho-3cDNA*, respectively. Only when all ‘fraction’ values in one dataset were ‘0.00,’ statistical analysis was not performed; in this case, we show a cross with colors, gray and black, representing transgenic animals with 5 ng/μL or 50 ng/μL, respectively. In (C, F), asterisks represent the comparison of transgenic animals with N2 animals by Dunnett test; *P < 0.05; **P < 0.01; ns, not significant (*P* > 0.05).

We examined whether over-expression of AHO-3 in AWC can affect the thermotactic plasticity after conditioning at 23 °C and at 20 °C (Fig. 4A–F and Fig. S9 in Supporting Information). We expressed AHO-3 using two promoters, *odr-1* promoter for the expression in AWC and AWB and *str-1* promoter for AWB. After conditioned at 23 °C, starved animals carrying *odr-1p::aho-3* showed a tendency to migrate toward higher-temperature region (Fig. 4A,C). Starved animals carrying *odr-1p::aho-3* conditioned at 20 °C showed a tendency to accumulate to near their cultivation temperature (Fig. 4D,F). The animals carrying *str-1p::aho-3*, however, showed no significant difference from wild-type animals both after conditioned at 23 °C and at 20 °C (Fig. 4B,C,E,F). These results suggest that the excess AHO-3 in AWC can affect the thermotaxis plasticity both after cultivation at 23 °C and at 20 °C.

**Figure 5:**
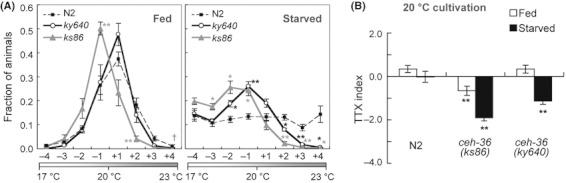
*ceh-36* mutants showed the abnormal thermotactic plasticity. (A, B) Thermotaxis of wild-type, *ceh-36(ks86)* and *ceh-36(ky640)* mutant animals cultivated at 20 °C with or without food. *n* ≥ 3 assays. Error bars represent SEM. In (A), asterisks represent the comparison of values in individual eight regions by unpaired *t*-test with the Dunn–Sidak correction for multiple comparisons; *P < 0.05; **P < 0.01; colors of asterisks, gray and black, represent the comparisons of N2 animals with each mutant, *ceh-36(ks86)* and *ceh-36(ky640)*, respectively. Only when all ‘fraction’ values in one dataset were ‘0.00,’ statistical analysis was not performed; in this case, we show a cross for *ceh-36(ks86)*. In (B), statistical significance of TTX indices was tested by unpaired *t*-test in comparisons of N2 animals with mutants; *P < 0.05; **P < 0.01.

To test whether AWC is required for the behavioral plasticity, we examined the thermotactic plasticity of *ceh-36(ks86)* and *ceh-36(ky640)* mutants that have a reduction in AWC function because of a mutation in an Otx-type homeobox gene required to specify the AWC cell fate (Lanjuin *et al.* 2003; Koga & Ohshima 2004). Well-fed *ceh-36* mutants showed a tendency to accumulate to the cultivation temperature, and starved *ceh-36* mutants showed cryophilic phenotype after cultivation at 20 °C (Fig. 5A,B). These results suggest that the lack of AWC function causes the cryophilic phenotype in thermotactic plasticity. Thus, AWC may induce thermophilic drive or suppress cryophilic drive after starvation. The double-mutant analyses showed that the *ceh-36(ky640)* mutation at least partially suppressed the abnormal thermophilic phenotype of *aho-3(nj15)* mutants after starvation (Fig. S10A,B in Supporting Information). Altogether, our results suggest that AHO-3 in AWC plays a role in thermotactic plasticity.

### *aho-3* acts in the same genetic pathway with *odr-3* for thermotactic plasticity

Genetic studies have identified several molecular components required for thermotactic plasticity. *ins-1* mutants, deficient in the insulin homologue, exhibit a defect in thermotactic plasticity (Kodama *et al.* 2006). INS-1 antagonizes DAF-2 insulin-like signaling, and this signaling pathway functions in thermotaxis interneurons (Kodama *et al.* 2006). Similarly, TAX-6 calcineurin was proposed to act downstream of DAF-2 in the thermotaxis interneurons (Kodama *et al.* 2006; Kuhara & Mori 2006). However, no one has reported genes that function in sensory neurons for thermotactic plasticity after starvation, although there are several other genes whose functional sites were not determined, for example, *gcy-28* gene coding receptor-type guanylyl cyclase whose mutations cause a defect in thermotactic plasticity (Tsunozaki *et al.* 2008).

Our results on the cell-specific rescue, over-expression and double-mutant analyses suggest that AHO-3 acts in sensory neurons including AWC for thermotactic plasticity (Figs 3C,D, 4A–F and 5A,B and Fig. S10A,B in Supporting Information). AWC is capable of sensing distinct stimuli, odor and temperature (Bargmann 2006; Biron *et al.* 2008; Kuhara *et al.* 2008). Several molecules have been reported to be important for AWC function. Guanylyl cyclase ODR-1 and G-alpha subunit ODR-3 act in chemotaxis and thermotaxis in AWC (Roayaie *et al.* 1998; L’Etoile & Bargmann 2000; Kuhara *et al.* 2008), cGMP-dependent protein kinase EGL-4 plays an important role in olfactory plasticity in AWC (L’Etoile *et al.* 2002; O’Halloran *et al.* 2009), and the GCY-28 also functions in AWC for the regulation of odor preferences (Tsunozaki *et al.* 2008).

**Figure 6:**
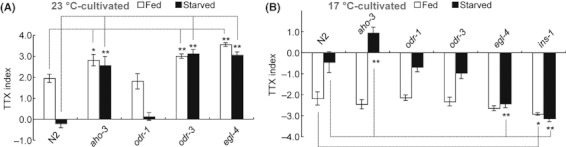
Thermotactic plasticity of mutants that have defects in AWC properties. (A, B) Thermotaxis of well-fed or starved wild-type N2 and mutant animals cultivated at 23 °C (A) or at 17 °C (B). *n* ≥ 3 assays. Statistical significance of TTX indices was tested by Dunnett test in comparisons of N2 animals with mutants; *P < 0.05; **P < 0.01. In (B), the results of N2 animals and *aho-3(nj15)* mutants were repeated from Figure 1B–D. *ins-1(nr2091)* mutants were assayed as control mutants that have defect in thermotactic plasticity after conditioned at 17 °C (Kodama *et al.* 2006).

To identify candidates that are relevant to AHO-3 function in thermotactic plasticity, we examined the thermotaxis of mutants deficient in AWC function. Fed and starved *odr-1(n1933)* mutants showed almost normal migration and dispersion after conditioning at both 23 and 17 °C (Fig. 6A,B). In contrast, although *odr-3(n1605)* mutants showed almost normal thermotactic plasticity after conditioning at 17 °C, they showed abnormal thermotactic plasticity after conditioning at 23 °C; starved *odr-3(n1605)* mutants conditioned at 23 °C migrated to higher-temperature region, similar to *aho-3(nj15)* mutants (Fig. 6A,B). *egl-4(n479)* mutants showed an abnormal thermotactic plasticity after conditioning at both 23 and 17 °C; starved *egl-4(n479)* mutants conditioned at 23 or at 17 °C migrated to higher- or lower-temperature regions, respectively, which roughly coincide with the cultivation temperature (Fig. 6A,B). These results suggest that the ODR-3 and EGL-4 are necessary for thermotactic plasticity. Abnormality of *odr-3(n1605)* and *egl-4(n479)* mutants each was partially rescued by expressing respective cDNA under the control of *odr-1* promoter (Fig. S11A,B in Supporting Information), implying that the ODR-3 and EGL-4 function in the thermotactic plasticity either or in both AWC and AWB.

**Figure 7:**
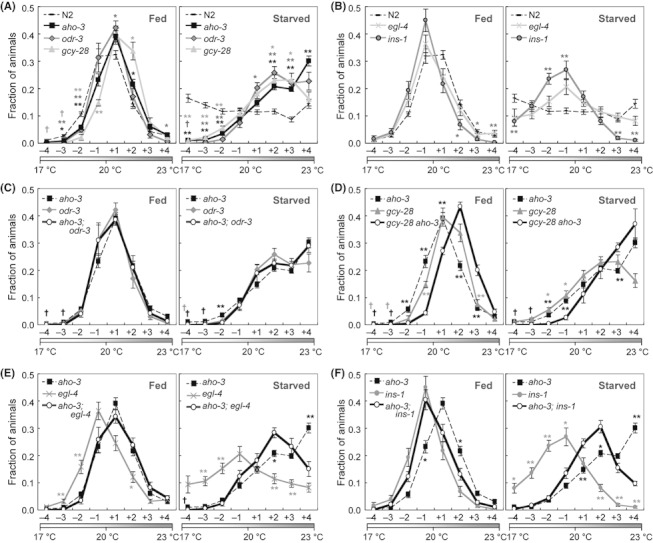
Genetic relationship analysis of *aho-3* with genes whose defects cause abnormal thermotactic plasticity. (A, B) Thermotaxis of wild-type N2 and single-mutant animals cultivated at 20 °C with or without food. Phenotypes of *aho-3(nj15)*, *odr-3(n1605)* and *gcy-28(tm2411)* are shown in (A), and those of *egl-4(n479)* and *ins-1(nr2091)* are shown in (B). (C–F) Thermotaxis of double and single mutants cultivated at 20 °C with or without food. Results of single mutants repeated from (A, B). In all graphs, asterisks represent the comparison of values in individual eight regions by unpaired *t*-test with the Dunn–Sidak correction for multiple comparisons; *P < 0.05; **P < 0.01; N2 animals vs (A, B) or double mutants vs (C–F) each single mutant. Only when all ‘fraction’ values in one dataset were ‘0.00,’ statistical analysis was not performed; in this case, we show a cross with colors, gray and black, representing single mutants and double mutants, respectively. *n* ≥ 3 assays. Error bars represent SEM.

To investigate the genetic relationships between *aho-3* and other genes such as *odr-3*, *egl-4*, *gcy-28* and *ins-1* that are required for thermotactic plasticity, we constructed double mutants and examined their thermotactic plasticity after cultivation at 20 °C (Fig. 7A–F and Fig. S12A–H in Supporting Information). Similar to *aho-3(nj15)* mutants, both *odr-3(n1605)* putative null mutants (Roayaie *et al.* 1998) and *gcy-28(tm2411)* strong loss-of-function mutants (Tsunozaki *et al.* 2008) cultivated under starved condition migrated to higher-temperature region (Fig. 7A and Fig. S12H in Supporting Information). We found that the thermotaxis of starved *aho-3;odr-3* double mutants was similar to that of each starved single mutants (Fig. 7C and Fig. S12H in Supporting Information). We also found that starved *gcy-28 aho-3* double mutants exhibited slightly enhanced thermophilic phenotype as compared with each single mutants (Fig. 7D and Fig. S12H in Supporting Information), and starved *gcy-28;odr-3* double mutants showed similar thermotaxis to each single mutants (Fig. S12A,H in Supporting Information). Behavioral phenotypes of these single and double mutants suggest that *aho-3*, *odr-3* and possibly *gcy-28* might act in the same genetic pathway to regulate the thermotactic plasticity.

Different from *aho-3(nj15)* mutants, a substantial proportion of starved *egl-4(n479)* and *ins-1(nr2091)* putative null mutants (Pierce *et al.* 2001; Fujiwara *et al.* 2002) migrated to their cultivation temperature (Fig. 7B). We found that starved *aho-3;egl-4* and *aho-3;ins-1* double mutants each showed thermotactic phenotype roughly intermediate between that of starved *aho-3(nj15)* single mutants and of starved *egl-4(n479)* or *ins-1(nr2091)* single mutants, respectively (Fig. 7E,F and Fig. S12H in Supporting Information). Similarly, starved double mutants, *egl-4;odr-3*, *ins-1;odr-3*, *gcy-28;egl-4* and *gcy-28;ins-1*, each showed thermotactic phenotype roughly intermediate between that of respective single mutants (Fig. S12C–F,H in Supporting Information). These results suggest that *aho-3*, *odr-3* and *gcy-28* at least partially act in parallel with *egl-4* and *ins-1*.

We also examined the thermotactic plasticity of three *aho-3;odr-3*, *gcy-28 aho-3* and *aho-3;egl-4* double mutants cultivated at 23 °C (Fig. S13A–G in Supporting Information). Phenotype of *aho-3;odr-3* mutants was similar to that of each single mutants (Fig. S13C,G in Supporting Information). Correspondingly, *gcy-28 aho-3* double mutants showed similar phenotype to *gcy-28* single mutants (Fig. S13D,G in Supporting Information). *aho-3;egl-4* double mutants, however, exhibited remarkably enhanced thermophilic phenotype in thermotactic plasticity (Fig. S13E,G in Supporting Information). Together with analyses of mutants cultivated at 20 °C, these results suggest that *aho-3* functions in the same genetic pathway with *odr-3* and *gcy-28*, whereas *aho-3* and *egl-4* act in parallel.

It was previously shown that *odr-3* encoding G-alpha functions downstream of *eat-16* encoding a regulator of G protein signaling protein (RGS) (Kuhara *et al.* 2008). The loss of EAT-16 causes a hyperactivation of AWC, leading to a cryophilic phenotype (Kuhara *et al.* 2008). In order to analyze the genetic relationship between *aho-3* and *odr-3* further, we examined the thermotactic plasticity of *aho-3 eat-16* and *eat-16;odr-3* double mutants after cultivation at 20 °C (Fig. S14A–E in Supporting Information). Both well-fed and starved *eat-16(nj8)* nearly null mutants (Kuhara *et al.* 2008) showed a cryophilic phenotype (Fig. S14A,D,E in Supporting Information). *odr-3(n1605)* mutation partially suppressed this phenotype (Fig. S14C–E in Supporting Information). Similarly, *aho-3(nj15)* mutation also showed partial suppression of the *eat-16(nj8)* phenotype (Fig. S14B,D,E in Supporting Information). Altogether, these results imply that, like *odr-3*, *aho-3* acts downstream of *eat-16*, although it is also implied that *eat-16* at least partially acts in parallel with *aho-3* and *odr-3*.

### The predicted catalytic triad and the N-terminal cysteine cluster are essential for AHO-3 function

The AHO-3 protein is highly conserved among animal species and possesses an alpha/beta-hydrolase domain at its C-terminus and a cysteine cluster in N-terminus. Although a few molecular properties of AHO-3 homologues have been previously characterized (Blankman *et al.* 2007; Kang *et al.* 2008; Martin & Cravatt 2009; Bachovchin *et al.* 2010; Marrs *et al.* 2010), biological functions *in vivo* have not been well understood. We analyzed the functional significance of domains of the AHO-3 protein for thermotactic plasticity in *C. elegans*, using recombinant AHO-3 proteins containing respective mutations.

Mouse AHO-3 homologues were identified as metabolic serine hydrolases (Blankman *et al.* 2007; Bachovchin *et al.* 2010). The metabolic serine hydrolases include esterases, lipases, peptidases and amidases (Holmquist 2000; Simon & Cravatt 2010). The majority of these enzymes use an alpha/beta-hydrolase fold and use a Ser–His–Asp catalytic triad (Holmquist 2000; Simon & Cravatt 2010). AHO-3 homologues also possess an alpha/beta-hydrolase domain including catalytic triad predicted by sequence comparison method (Fig. 2C and Fig. S5 in Supporting Information; UniProtKB, http://www.uniprot.org/uniprot/Q5VST6; merops database, http://merops.sanger.ac.uk) (Rawlings *et al.* 2010).

**Figure 8:**
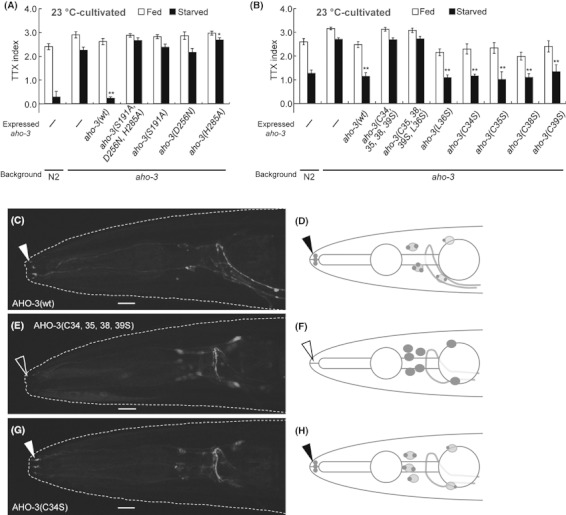
Predicted catalytic residues and the N-terminal cysteine cluster in AHO-3 are essential for thermotactic plasticity. (A, B) Rescue experiment for the abnormal thermotactic plasticity of *aho-3(nj15)* mutants with recombinant AHO-3::EGFP containing a mutation in the predicted catalytic residues (A) or into the N-terminal cysteines (B). Recombinant proteins were expressed pan-neuronally. Thermotaxis of well-fed or starved animals cultivated at 23 °C. *n* ≥ 3 assays. Error bars represent SEM. Asterisks represent the comparison of starved transgenic animals with starved *aho-3* mutants by Dunnett test; *P < 0.05; **P < 0.01.(C–H) Subcellular localization of AHO-3(wild-type)::EGFP (C), AHO-3(C34, 35, 38, 39S)::EGFP (E) and AHO-3(C34S)::EGFP (G) expressed under the control of the *aho-3* promoter in adult *aho-3(nj15)* mutants. Solid arrowheads point to the localization of AHO-3(wild-type)::EGFP and AHO-3(C34S)::EGFP to sensory endings (C and G). AHO-3(C34, 35, 38, 39S)::EGFP was localized diffusely in cell bodies and not to sensory endings (open arrowhead; E). Schematic diagrams of (C), (E) and (G) are shown in (D), (F) and (H), respectively. Images are Z-stack confocal projection. A rough outline of head of animal is shown (C, E and G). Anterior is to the left. Bars represent 10 μm.

To determine whether predicted catalytic triad of AHO-3, Ser 191, Asp 256 and His 285, is required for thermotactic plasticity, we constructed mutant *aho-3* genes containing mutations that disrupt the probable Ser–His–Asp catalytic triad and expressed it pan-neuronally in *aho-3(nj15)* mutants (Fig. S15A–E in Supporting Information). The abnormal thermotactic plasticity of *aho-3(nj15)* mutants was rescued by expressing wild-type AHO-3, but not by mutant AHO-3 proteins (S191A, D256N and/or H285A) (Fig. 8A). These results suggest that the predicted catalytic residues are essential for AHO-3 function in thermotactic plasticity and that AHO-3 acts as an enzyme in this behavioral modification.

Rat AHO-3 homologues were previously identified as a protein modified by a fatty acid palmitate (Kang *et al.* 2008). Protein palmitoylation is a post-translational modification in which 16-carbon fatty acid palmitate is added to specific cysteine residues (Linder & Deschenes 2007; Fukata & Fukata 2010). Other group discovered that the human AHO-3 homologues are palmitoylated at the N-terminal cysteine cluster (Martin & Cravatt 2009), which is conserved among animal species (Fig. 2C and Fig. S5 in Supporting Information), and that this cluster is necessary for their own localization to plasma membrane (Martin & Cravatt 2009).

We analyzed the subcellular localization of AHO-3 by expressing recombinant AHO-3 proteins fused with fluorescent proteins under the control of the *aho-3* promoter. The AHO-3::EGFP expression by the *aho-3* promoter rescued the abnormal thermotactic plasticity of *aho-3(nj15)* mutants (Fig. 3C). These AHO-3::EGFP proteins were observed in sensory endings, cell bodies as punctiform and often the nerve ring (Fig. 8C,D and Fig. S16A–F in Supporting Information). The punctate stainings of the AHO-3 protein in cell bodies were observed very near to the stainings of Golgi markers, mannosidase::YFP and MIG-23::GFP (Rolls *et al.* 2002; Nishiwaki *et al.* 2004) (Fig. S16A–L in Supporting Information). We examined whether the subcellular localization is changed depending on the feeding states, but there was no apparent difference between fed and starved animals under the 23 °C cultivation (Table S3 in Supporting Information).

To analyze whether the N-terminal cysteine cluster of AHO-3 is required for its localization, we constructed translational AHO-3::EGFP containing mutations in the N-terminal cysteines (Cys 34, 35, 38 and 39) and generated transgenic animals. The recombinant AHO-3(C34, 35, 38 and 39S)::EGFP was localized diffusely in cell bodies and not to sensory endings (Fig. 8E,F and Table 1). Another mutant AHO-3(C34S)::EGFP containing a mutation in only one cysteine was localized to sensory endings and cell bodies in punctiform, although it seemed to be moderately diffused (Fig. 8G–H and Table 1). These results suggest that the N-terminal cysteine cluster of AHO-3 is necessary for its subcellular localization and that a certain number of cysteines may be essential for its localization to sensory endings.

**Table 1:** Subcellular localization analysis of recombinant AHO-3

Subcellular location	Recombinant AHO-3	Category AHO-3 fluorescence			Total *n*

		Strong	Weak	Invisible	
Sensory endings	Wild type	60	17	3	80
	C34S	65	17	2	84
	C34, 35, 38, 39S**	18	24	42	84
Punctuations in cell bodies	Wild type	62	14	4	80
	C34S**	2	37	45	84
	C34, 35, 38, 39S**	0	0	84	84
Diffusion in cell bodies	Wild type	4	56	20	80
	C34S**	42	35	7	84
	C34, 35, 38, 39S**	61	23	0	84

* *P* < 0.01.

Localization of AHO-3 was evaluated in adults carrying each types of *aho-3p::aho-3cDNA::egfp*. Animals were cultivated at 20 °C with food. We categorized fluorescence intensity in sensory endings and cell bodies into strong, weak and invisible. Statistical analysis by a chi-square test using a 2 × 3 contingency table was performed to compare the AHO-3(wild type) with each recombinant AHO-3.

We also examined the requirement of the N-terminal cysteine cluster of AHO-3 for thermotactic plasticity. The abnormal thermotactic plasticity of *aho-3(nj15)* mutants was not rescued by pan-neuronal expression of the recombinant AHO-3(C34, 35, 38 and 39S)::EGFP or the recombinant AHO-3(C35, 38 and 39S, L36S)::EGFP keeping only one cysteine, whereas the abnormality was fully rescued by expressing the recombinant AHO-3(C34, 35, 38 or 39S)::EGFP keeping three cysteines (Fig. 8B and Fig. S17A–F in Supporting Information). These results suggest that the N-terminal cysteine cluster of AHO-3 is necessary and a certain number of cysteines in the cluster are essential for thermotactic plasticity. Altogether, these results suggest that the proper subcellular localization of AHO-3 to sensory endings is crucial for thermotactic plasticity.

## Discussion

We here reported that the highly conserved novel hydrolase AHO-3 is required for *C. elegans* behavioral plasticities. Rescue experiments suggest that the molecular function of the AHO-3 protein is conserved between nematode and human. We propose that the AHO-3 protein plays conserved important roles in neural function across animal species.

### The function of AHO-3 in thermotactic plasticity in *Caenorhabditis elegans*

It was previously shown that *C. elegans* exhibits thermotactic plasticity; well-fed animals migrate to their cultivation temperature, and starved animals avoid the cultivation temperature on the nonlinear thermal gradient (Mohri *et al.* 2005). Our study showed that starved animals *disperse* on the linear thermal gradient except for the 20 °C-cultivated starved animals assayed in the thermal gradient ranging from 20 to 26 °C: these animals migrated to the colder region. These different results can be caused by the differences in the steepness of the thermal gradients or starting-point temperature, because variations in these factors affect the thermotaxis of well-fed animals (Ramot *et al.* 2008; Nakazato & Mochizuki 2009; Jurado *et al.* 2010; Beverly *et al.* 2011). In all of these assays, however, animals modify their behavior after starvation. We call all forms of these plasticities ‘thermotactic plasticity’ in this article.

*aho-3* mutants exhibit abnormality in thermotactic plasticity. Like wild-type animals, well-fed *aho-3* mutants migrated to the cultivation temperature on a thermal gradient (Fig. 1B,E,H and Fig. S1A,D,G in Supporting Information). Whereas starved wild-type animals showed dispersed distribution or migration to colder region, starved *aho-3* mutants exhibited tendency to migrate toward higher temperature than the starved wild-type animals (Fig. 1C,F,I and Fig. S1B,E,H in Supporting Information). The simplest model for thermotaxis in *C. elegans* is that the behavior is a result of balanced regulation of two opposing thermophilic and cryophilic drives (Hedgecock & Russell 1975). Based on this model, we could assume that starvation conditioning changes the strength of these opposing drives. Considering the behavioral abnormality of *aho-3* mutants, it is likely that AHO-3 protein suppresses the thermophilic drive or promotes the cryophilic drive in response to the starvation signal.

Our cell-specific rescue experiments showed that AHO-3 functions in many sensory neurons including the AWC thermosensory neuron for thermotactic plasticity (Fig. 3C,D). The AHO-3 expression in both AWC and AWB neurons partially rescued the abnormality of *aho-3* mutants after cultivation at 20 and 23 °C (Fig. 3C,D). The expression in only AWC partially rescued the abnormality after cultivation at 23 but not at 20 °C (Fig. 3C,D). The over-expression analysis, however, showed that the excess AHO-3 in AWC but not in AWB caused an abnormal thermotactic plasticity both after cultivation at 20 °C and at 23 °C (Fig. 4A–F). In addition, the reduction in AWC function partially suppressed the defect of *aho-3* mutants after cultivation at 20 °C (Fig. S10A,B in Supporting Information). Altogether, our results suggest that AHO-3 acts in AWC for thermotactic plasticity, although the AHO-3 activity in other neurons is also important.

It was reported that AWC regulates the activity of downstream AIY interneuron that promotes thermophilic drive (Kuhara *et al.* 2008; Ohnishi *et al.* 2011). Our study suggested that AWC is important for thermotactic plasticity (Fig. 5A,B), but the calcium-imaging analysis did not detect any significant difference in the responses of AWC or AIY of fed and starved animals (Fig. S18A–G in Supporting Information). The changes in AWC or AIY activity after starvation may be too subtle to detect.

Double-mutant analyses suggested that *aho-3* acts in the same genetic pathway with *odr-3* encoding G protein alpha subunit (Fig. 7C and Figs S12H, S13C and S13G in Supporting Information). ODR-3 is required for thermosensation in AWC and is localized to sensory endings (Roayaie *et al.* 1998; Bargmann 2006; Kuhara *et al.* 2008). Our results showed that AHO-3 localized to sensory endings like ODR-3 (Fig. 8C). These results imply that AHO-3 acts in the ODR-3-mediated G protein signaling pathway to inhibit the thermophilic drive after starvation in AWC.

Our over-expression experiments, however, showed that the excess AHO-3 in AWC did not simply induce cryophilic phenotype (Fig. 4A,C,D,F). Because AWC transmits both excitatory and inhibitory signals to AIY (Kuhara *et al.* 2008; Ohnishi *et al.* 2011), it is possible that AHO-3 modifies these AWC transmissions in complex manner, regulating thermotactic plasticity after starvation.

### Molecular function of the highly conserved AHO-3 protein across animal species

The novel hydrolase AHO-3 is highly conserved from flat animals to human (47%–66% amino acid sequence identity and 61%–80% similarity; Fig. 2B–D, Figs S5 and S6 in Supporting Information). Previous studies showed that mammalian AHO-3 homologues are expressed in the brain (Bachovchin *et al.* 2010) (EMBL-EBI, http://www.ebi.ac.uk/gxa/). Rescue experiment with human homologue of AHO-3 suggests that molecular properties of AHO-3 are conserved between nematode and human (Fig. 2E). Although *in vivo* functions of AHO-3 homologues have remained unknown, a few molecular properties have been evaluated (Blankman *et al.* 2007; Kang *et al.* 2008; Martin & Cravatt 2009; Bachovchin *et al.* 2010; Marrs *et al.* 2010).

The activity-based profiling showed that mouse AHO-3 homologues belong to the metabolic serine hydrolase superfamily (Blankman *et al.* 2007; Bachovchin *et al.* 2010), whose members mostly possess an alpha/beta-hydrolase domain including a catalytic triad (Holmquist 2000; Simon & Cravatt 2010). All AHO-3 homologues also have an alpha/beta-hydrolase domain including predicted catalytic triad (Fig. 2C, Fig. S5 in Supporting Information), which is required for AHO-3 function in thermotactic plasticity (Fig. 8A). It is yet unknown what kind of substrates AHO-3 homologues react to. So far, only one of the AHO-3 similar proteins, ABHD12 (see Fig. 2D), was suggested to hydrolyze 2-arachidonoylglycerol, an endogenous ligand for cannabinoid receptors, in mouse brain (Blankman *et al.* 2007; Marrs *et al.* 2010). It is possible that AHO-3 proteins play a role in the degradation of small neural molecules, for example some sorts of ligand or intracellular messenger.

Human AHO-3 homologues were identified as a protein modified by a fatty acid palmitate (Martin & Cravatt 2009). Palmitoylation is a unique lipid-based post-translational modification in that it is reversible (Linder & Deschenes 2007; Fukata & Fukata 2010). The reversible nature allows palmitoylation to regulate diverse aspects of neuronal protein trafficking, localization and functions related to neurite outgrowth and neural plasticity (Linder & Deschenes 2007; Fukata & Fukata 2010). N-terminal cysteine cluster, the putative palmitoylation motif in human AHO-3 homologues (Martin & Cravatt 2009), is conserved in all AHO-3 homologues (Fig. 2B,C and Fig. S5 in Supporting Information). We showed here that the putative palmitoylation motif of *C. elegans* AHO-3 is necessary for its localization to sensory endings and for thermotactic plasticity (Fig. 8B–H). Given the property of palmitoylation, our results imply that palmitoylation may regulate the AHO-3 localization that is necessary for its function and consequently modify multiple behaviors such as thermotactic plasticity. Further molecular, biochemical and behavioral analyses on AHO-3 using *C. elegans* and other model animals should predict its functions in the nervous system and general roles in behavioral modifications through animal species.

## Experimental procedures

### Strains and maintenance

*Caenorhabditis elegans* strains were maintained with *E. coli* OP-50 and handled according to standard procedures (Brenner 1974). We used the following strains: wild-type Bristol strain (N2), wild-type Hawaiian strain (CB4856) for mapping with the snip-SNPs method, IK850 *aho-3(nj15) I* backcrossed 10 times with N2 (six times by phenotype of abnormal thermotactic plasticity and four times by *nj15* genotype), IK849 *aho-3(nj15) I* backcrossed six times with N2 only for mapping in Figure 2A and Figure S4 in Supporting Information and for behavioral tests in Figure S2E–H in Supporting Information, JC2154 *hen-1(tm501) X*, MT3644 *odr-3(n1605) V*, IK852 *gcy-28(tm2411) I*, IK571 *egl-4(n479) IV*, IK607 *ins-1(nr2091) IV*, CX2349 *odr-1(n1933) X*, *ceh-36(ks86) X*, *ceh-36(ky640) X*, *eat-16(nj8) I*, *aho-3(nj15) I; odr-3(n1605) V*, *gcy-28(tm2411) aho-3(nj15) I*, *gcy-28(tm2411) I; odr-3(n1605) V*, *aho-3(nj15) I; egl-4(n479) IV*, *egl-4(n479) IV; odr-3(n1605) V*, *gcy-28(tm2411) I; egl-4(n479) IV*, *aho-3(nj15) I; ins-1(nr2091) IV*, *ins-1(nr2091) IV; odr-3(n1605) V*, *gcy-28(tm2411) I; ins-1(nr2091) IV*, *egl-4 (n479) ins-1 (nr2091) IV*, *aho-3(nj15) eat-16(nj8) I*, *eat-16(nj8) I; odr-3(n1605) V*, *aho-3(nj15) I; ceh-36(ky640) X* and transgenic strains derived from them. All transgenic strains were made essentially as described (Mello *et al.* 1991), with the co-injection marker *ges-1p::NLS-GFP* (pKDK66) for the rescue experiment strains and over-expression experiment strains, *rol-6gf* (pRF4) or *ges-1p::NLS-TagRFP* (pNAS88) for the subcellular localization test strains, and *rol-6gf* (pRF4; for Fig. S7B,O–Q in Supporting Information), *ges-1p::NLS-GFP* (pKDK66; for Fig. S7R in Supporting Information) or none (for Fig. 3A, Fig. S7A,C–N in Supporting Information) for the expression pattern test strains. Test plasmids were injected at 2–50 ng/μL. In Figure 3C,D, rescue experiment strains carried *aho-3 cDNA* fused with each cell-specific promoter, except for *aho-3p* and *ceh-36p* fused with *aho-3 cDNA::egfp*. In Figure S11A in Supporting Information, *odr-3(n1605); Ex[odr-1p::odr-3 cDNA]* was made by outcrossing with N2; *Ex[odr-1p::odr-3 cDNA]* (Kuhara *et al.* 2008). At least two independent lines were tested for each rescue and over-expression experiment, except for *unc-14p::aho-3 cDNA* and *odr-1p::aho-3 cDNA* in Figure 3D and Figure S8A–C and for genomic PCR fragment in Figure S2B,D and S3A–D in Supporting Information. We tested the same one line in Figure S2B,D and S3A–D in Supporting Information.

### Behavioral analysis

#### The population thermotaxis assay

The population thermotaxis assay was performed as previously reported (Ito *et al.* 2006) with some modifications according to Mohri *et al.* (Mohri *et al.* 2005) for evaluating thermotactic plasticity associated with feeding states. Equipment for establishing the linear thermal gradient was used as described (Hedgecock & Russell 1975; Ito *et al.* 2006). A stable, linear thermal gradient was established on a 60-cm-long aluminum platform by using two water baths at 5 and 35 °C. TTX (thermotaxis) plate (13.5 cm × 6 cm, 1.8 cm height) containing 10 mL of TTX medium (2% agar, 0.3% NaCl, 25 mm potassium phosphate, pH 6.0) was placed on the platform. The extra space between the bottom of the TTX plate and the platform was filled with water to increase the thermal conductivity as much as possible. Except for Figure S1 in Supporting Information, the center of the 13.5-cm-long agar surface in TTX plate was adjusted at 20 °C, and linear thermal gradient ranging from 17 to 23 °C was established on the agar surface. In Figure S1 in Supporting Information, the center of TTX plate was adjusted at 23 °C, and linear thermal gradient from 20 to 26 °C was established.

We cultivated animals under uncrowded and well-fed condition at respective temperatures, 17, 20 or 23 °C, on a 6-cm plate containing 14 mL of nematode growth medium (NGM) with 2% agar, on which *E. coli* OP-50 was seeded. Except for Figures 1B–D and 6B, well-fed naive animals were collected and washed twice with NG buffer (0.3% NaCl, 1 mm CaCl_2_, 1 mm MgSO_4_, 25 mm potassium phosphate, pH 6.0) in population thermotaxis assay with well-fed animals. This NG buffer was pre-incubated and kept at 20 °C (except for Fig. S1 in Supporting Information) or at 23 °C (only for Fig. S1 in Supporting Information). Approximately 40–300 animals were placed at the center of the TTX plate, and excess water was removed with tissue paper immediately. The TTX plates were left undisturbed and animals were allowed to move freely for 60 min. After animals were killed by chloroform gas or immobilized with ice, the adult animals in each of the eight regions were counted. The TTX index, defined as shown in Figure 1A, was calculated. In population thermotaxis assay with starved animals, the well-fed naive animals were collected and washed twice with NG buffer and placed on starvation-conditioning plate (2% agar, 1 mm CaCl_2_, 1 mm MgSO_4_, 25 mm potassium phosphate, pH 6.0) without food; those NG buffer and starvation-conditioning plates were pre-incubated and kept at each cultivation temperature. Excess water was removed immediately after placement. After cultivation without food for designated time (see below), animals were collected with NG buffer kept at 20 °C (except for Fig. S1 in Supporting Information) or at 23 °C (only for Fig. S1 in Supporting Information), and approximately 40–300 animals were placed at the center of the TTX plate. The subsequent procedure was the same as described for the well-fed animals. In Figures 1B–D and 6B, we performed population thermotaxis assay almost similar to described above, except that animals were picked instead of washed when they were transferred from a plate onto other plate (e.g., from NGM plate onto starvation-conditioning plate) and that 18–55 animals were placed on the TTX plate. Starvation-conditioning plates contained a high-osmolarity ring of 8 m glycerol at the periphery in Figures 1B–D and 6B to prevent the animals from swimming off the agar; the 8 m glycerol ring does not affect thermotaxis (Mohri *et al.* 2005). In this study, we designated starvation time 3 h at 17 °C, 2 h at 20 °C and 1 h at 23 °C, respectively, because it was reported that behavioral change induced by starvation sufficiently occurs after cultivation at 17 °C for 3 h or after cultivation at 25 °C for 1 h (Mohri *et al.* 2005).

#### Salt chemotaxis learning assay

We performed salt chemotaxis learning assay according to previous reports (Tomioka *et al.* 2006) with some modifications. Test animals were cultivated under well-fed condition at 20 °C. We used ‘liquid conditioning method’ and assayed on 6-cm test plates with a salt gradient made by an agar plug containing 50 mm NaCl. The calculation of Chemotaxis index was modified as shown in Figure S2A in Supporting Information. Detailed methods are described in Supplementary materials.

#### Interaction assay, Chemotaxis assay and Avoidance assay

The procedure for interaction assay between chemotaxis to odorants and avoidance of Cu^2+^ ion was according to the previous report (Ishihara *et al.* 2002). 1/100 diacetyl and 100 mm Cu^2+^ ion were used. Test animals were cultivated under well-fed condition at 17, 20 or 25 °C. The procedures for assaying chemotaxis to volatile odorant and assaying avoidance from Cu^2+^ ion were according to Bargmann *et al.* (Bargmann *et al.* 1993) and Wicks *et al.* (Wicks *et al.* 2000), respectively. Test animals were cultivated under well-fed condition at 25 °C.

### Molecular biology

*aho-3* cDNA was amplified from yk1293d3 and cloned into pPD49.26 to generate pNAS1 using the Acc65I and EcoRI sites. *aho-3* cDNA from pNAS1 was inserted into each promoter construct to generate specific promoter::*aho-3* cDNA plasmids. Specific promoters are *unc-14p* for pan-neuronal (Ogura *et al.* 1997), *osm-6p* for ∼20 pairs of sensory neurons (Collet *et al.* 1998; Kodama *et al.* 2006), *ncs-1p* for AIY and ∼10 pairs of sensory neurons (Gomez *et al.* 2001), *glr-1p* for ∼15 pairs of interneurons (Hart *et al.* 1995; Maricq *et al.* 1995), *glr-2p* for ∼10 pairs of interneurons (Brockie *et al.* 2001), *odr-1p* for AWC and AWB (L’Etoile & Bargmann 2000), *ceh-36prom3* for AWC (Etchberger *et al.* 2007), *gcy-8p* for AFD (Inada *et al.* 2006), *ttx-3p* for AIY (Kodama *et al.* 2006; Kuhara & Mori 2006), *tph-1p* for ADF, HSN and NSM (Sze *et al.* 2000), *srh-142p* for ADF (Sagasti *et al.* 1999; Chang & Bargmann 2008), *glr-3p* for RIA (Brockie *et al.* 2001) and *str-1p* for AWB (Troemel *et al.* 1997) (Table S2 in Supporting Information). The *aho-3* genomic sequence including 4 kb of the promoter region was amplified from the N2 genome by PCR and cloned into pBluescript II SK+ to generate pFUG2. The 4-kb promoter region of *aho-3* was amplified from pFUG2 and cloned into pPD95.75 or into pNAS1 to generate *aho-3p::gfp* (pNAS14) and *aho-3p::aho-3 cDNA* (pNAS15) using the BamHI site. We cloned *aho-3* cDNA amplified from *unc-14p::aho-3cDNA* (pNAS3) and *EGFP* amplified from *ttx-7::EGFP* (Tanizawa *et al.* 2006) (generated from pEGFP-N1; Takara Bio) into pNAS3 to generate *unc-14p::aho-3 cDNA::EGFP* (pNAS42); *aho-3 cDNA* and *EGFP* was fused with NsiI site. *aho-3p::aho-3 cDNA::EGFP* (pNAS50) was generated from pNAS15 and pNAS42 using the Acc65I, EcoRI and PvuI sites. *aho-3p::aho-3 cDNA::CFP* (pNAS141) and *aho-3p::aho-3 cDNA::DsRed-monomer* (pNAS140) were constructed from pNAS50; *aho-3 cDNA* and the marker genes were fused with NsiI sites. FAM108B1 cDNA was amplified from EHS1001-10687 (Open Biosystems, Inc.) and cloned to make *unc-14p::*FAM108B1 cDNA (pNAS72). We generated recombinant *aho-3* cDNAs, used in Figure 8 and Figures S15 and S17 in Supporting Information, by site-directed mutagenesis from pNAS3 or pNAS42 and constructed *unc-14p::aho-3 cDNA(recombinant)::EGFP*. From those plasmids containing *unc-14p* fusion recombinant *aho-3* cDNAs and from pNAS50, we constructed *aho-3p::aho-3 cDNA(recombinant)::EGFP* using the Acc65I and NsiI sites. *aho-3p::Mans::YFP* (pNAS132) was constructed from pNAS50 and *glr-3p::Mans::YFP* (pUBA23) using the SphI and Acc65I sites. *aho-3p::mig-23::GFP* (pNAS136) was constructed from pNAS50 and *mig-23::GFP* using the BamHI sites. *aho-3p::cytochrome b5::yfp* (pNAS128) and *odr-1p::cytochrome b5::yfp* (pNAS129) were constructed from pNAS50 or *odr-1p::aho-3 cDNA::egfp* (pNAS96) and from *glr-3p::cytochrome b5::yfp* using the SphI and Acc65I sites. *odr-1p::cytochrome b5::cfp* (pNAS158) was constructed from pNAS129 and from *AIYp::Mans::cfp* using the XmaI and AatII sites. *tph-1p::NLS-tagRFP* (pNAS160) was constructed from *tph-1p::GFP* (pOKU82) and pNAS88 using the SalI and StyI sites. *unc-14p::odr-3cDNA* (pNAS77) was generated from pNAS3 and *odr-3* cDNA. *unc-14p::egl-4.a cDNA* (pNAS85) and *odr-1p::egl-4.a cDNA* (pNAS99) were generated from *egl-4.a* cDNA vector and pNAS3 or *odr-1p::aho-3* cDNA (pNAS8), respectively.

### Sequence analysis of AHO-3

We used the Pfam program (http://www.sanger.ac.uk/resources/databases/pfam.html) and the sosui program (http://bp.nuap.nagoya-u.ac.jp/sosui/) to analyze the structure of the *C. elegans* AHO-3 protein, and it was predicted that the AHO-3 possesses an alpha/beta-hydrolase domain and no transmembrane segment. The sosui program was also used to predict the transmembrane segment of *S. cerevisiae* YNL320W in Figure 2B. Because extents of alpha/beta-hydrolase domains predicted from Pfam program were slightly different from one another in AHO-3 homologues and similar proteins, those extents of domains in Figure 2B are predicted from sequence alignment with the domain sequence in *C. elegans* AHO-3 protein, which was predicted from Pfam program. To search the AHO-3 homologues and AHO-3 similar proteins in 17 biological species, we use Web blast services provided by the Kyoto Encyclopedia of Genes and Genomes (http://www.genome.jp/kegg/), Joint Genome Institute (http://www.jgi.doe.gov/) and WormBase (http://www.wormbase.org/). In the human proteome, except for the FAM108 proteins, most similar proteins to *C. elegans* AHO-3 were ABHD12, ABHD12B and ABHD13, consistent with a previous report (Simon & Cravatt 2010) and a database (Tree families database, http://www.treefam.org/). Figure 2D and Figure S6 in Supporting Information show dendrograms of similar proteins to FAM108 proteins, ABHD12 proteins and ABHD13 protein in 11 animal species and 2 or 6 other species.

### Analyses of expression and localization of AHO-3

All of the fluorescence images were taken with a confocal laser scanning microscope Fluoview FV1000 (Olympus) except for the image in Figure S7R in Supporting Information, which was taken with an Axioplan2 light microscope (Zeiss). We identified the neurons expressing a reporter gene under the control of the *aho-3* promoter, based on their position and morphology or their co-expression with cell-specific markers, *odr-1p::cytochrome b5::cfp*, *gcy-8p::tagRFP*, *AIYp::tagRFP* and *tph-1p::NLS-tagRFP*. We observed and evaluated recombinant AHO-3::EGFP localization in head neurons of adult animals with an Axioplan2 light microscope in Table 1 and Table S3 in Supporting Information. Recombinant AHO-3::EGFP proteins were expressed under the control of the *aho-3* promoter in *aho-3(nj15)* mutants. Test animals were cultivated at 20 °C (Table 1) or at 23 °C (Table S3 in Supporting Information). We scored the fluorescence intensity in sensory endings, punctiform staining of cell bodies and entire cell bodies by using a three-point scale (strong, weak and invisible).

### *In vivo* calcium imaging

*In vivo* calcium imaging was performed essentially according to previous reports (Kuhara *et al.* 2008; Ohnishi *et al.* 2011). Detailed methods are described in Supporting Information.

### Statistics

All behavioral assays were performed at least three times in separate experiments. In all Figures, error bars represent standard error of mean (SEM). To compare the distributions of animals in thermotaxis assay, statistical significance of ‘fraction of animals’ in each region was tested by the unpaired *t*-test with the Dunn–Sidak correction for multiple comparisons. Only when all ‘fraction of animals’ values in one dataset were ‘0.00,’ statistical analysis was not performed; in this case, we show a cross in each Figures. To compare other values, statistical significance was tested by the unpaired *t*-test, Tukey’s test or Dunnett test.

In the localization analysis of AHO-3, more than 25 animals (Table 1) or 10 animals (Table S3 in Supporting Information) were tested at each trial in 3 days. We scored the fluorescence intensity by using a three-point scale, and statistical analysis by a chi-square test was performed to compare the wild-type AHO-3 with each mutated AHO-3 or the AHO-3 in fed animals with the AHO-3 in starved animals.

## Supporting Information/Supplementary material

The following Supporting Information can be found in the online version of the article:

**Appendix S1** Experimental procedures.

**Figure S1** Abnormal thermotactic plasticity of *aho-3* mutants on the 20–26 °C thermal gradient.

**Figure S2** *aho-3* mutants show defects in the salt learning behavior and integration behavior.

**Figure S3** Rescue experiment of *aho-3* mutants with K04G2.2 gene for the abnormal thermotactic plasticity.

**Figure S4** Rescue experiments to identify the gene responsible for *aho-3* mutants.

**Figure S5** Alignment of AHO-3 homologs and similar proteins.

**Figure S6** AHO-3 novel protein is highly conserved among animal species.

**Figure S7** A reporter gene expression under control of the *aho-3* promoter.

**Figure S8** Cell-specific rescue experiments for *aho-3* mutants conditioned at 20 °C.

**Figure S9** Locomotive ability of animals overexpressing AHO-3 in AWC.

**Figure S10** The analysis with *aho-3;ceh-36* double mutants.

**Figure S11** Rescue experiment for the abnormal thermotactic plasticity of *odr-3* and *egl-4* mutants.

**Figure S12** Genetic relationship analysis among genes whose defect cause abnormal thermotactic plasticity; for 20 °C-cultivation.

**Figure S13** Genetic relationship analysis of *aho-3* with *odr-3*, *gcy-28* and *egl-4*; for 23 °C-cultivation.

**Figure S14** Genetic relationship analysis among *aho-3*, *odr-3* and *eat-16*.

**Figure S15** Expressions and localizations of AHO-3 mutated in the predicted cataritic triad.

**Figure S16** Subcellular localization assay of AHO-3.

**Figure S17** Expressions and localizations of AHO-3 mutated in the N-terminal cysteines.

**Figure S18** *In vivo* calcium imagingof AWC and AIY according to temperature change in *aho-3* mutants.

**Table S1** AHO-3 homologs and similar proteins

**Table S2** Expression patterns driven by each promoter

**Table S3** Subcellular localization analysis of AHO-3 with fed and starved animals
