# Supplementary material for: A novel and conserved protein AHO-3 is required for thermotactic plasticity associated with feeding states in Caenorhabditis elegans
Source: Genes Cells. 2012 May;17(5):365–86. doi: 10.1111/j.1365-2443.2012.01594.x (PMC3506735; doi:10.1111/j.1365-2443.2012.01594.x)
Supplement: Supplementary file 1 [file gtc0017-0365-SD1.pdf]

Supporting Information

1. Supporting Experimental procedures

2. Supporting Figure Legends

3. Supporting Figures

4. Supporting Tables

Supporting Experimental procedures

Behavioral analysis

The individual thermotaxis (TTX) assay. We performed the individual TTX assay only for genetic mapping and for rescue experiments with genomic and PCR fragments to identify the gene responsible for the *aho-3(nj15)* mutant in Figure 2A and Figure S4. The individual TTX assay was performed same as described by Mohri *et al*. (2005). We used a 45 ml glass vial (Wheaton) containing frozen acetic acid for creating a stable radial thermal gradient on a TTX assay plate (9 cm in diameter); the center is approximately 17˚C and the periphery is approximately 25˚C. The L4 larvae grown under well-fed condition were cultured at 25˚C for 8–16 hours under uncrowded and well-fed condition, 12–40 animals per 6 cm NGM plate. Fully matured adult animals were transferred to an assay plate with radial thermal gradient and allowed to move freely for 50 minutes. For the assay with starved animals, well-fed animals were washed three times with NG buffer, transferred to the starvation-conditioning plates, cultured at 25˚C for one hour, and then assayed. Thermotaxis of individual animals was evaluated by using four phenotypic categories based on the tracks left on a assay plates; animals

that moved to the cold region were classified as '17', animals that moved to the 20˚C region were classified as '20', animals that moved to the warm region were classified as '25', and animals that moved to the cold and warm regions or moved like randomly were classified as '17/25' (Figure S4A).

Salt chemotaxis learning assay. This assay was performed as described (Tomioka *et al*., 2006) with some modifications. We performed chemotaxis assays on 6-cm assay plates containing 5 ml of medium (2% agar, 1 mM $CaCl_2$, 1 mM $MgSO_4$, 5 mM potassium phosphate, pH 6.0), on which a salt gradient had been formed for 18-22 hour by using an agar plug containing 50 mM NaCl. 1 ul of 0.5 M sodium azide was spotted at the gradient peak and at the opposite ends of the plates. Adult animals grown at 20˚C on NGM plates seeded with *E. coli* OP-50 were collected, washed twice, transferred to 2 ml of conditioning buffer (5 mM KPO4, pH 6.0, 1 mM CaCl2, 1 mM MgSO4) with 20 mM NaCl (NaCl conditioning) or without NaCl (mock conditioning), and incubated at 23˚C for 1 hour with gentle rotation. The buffer was pre-incubated and kept at 23˚C. After the incubation, animals were washed with 1-2 ml of mock-conditioning buffer and placed at the center of the assay plates. Excess water was removed with tissue paper immediately. Then, plates were left undisturbed at room temperature for 15 min. The chemotaxis index was calculated as (A — B)/(total) where A was the number of animals within 1 cm of the peak of the salt gradient, B was the number of animals within 1 cm of the control spot, "total" was the number of all animals on an assay plate except the number of animals that did not move in the central region (Figure S2A). 20 to 130 animals were used in each assay, and assays were independently performed at least four

times.

In vivo calcium imaging and data analysis

In vivo calcium imaging was performed essentially according to previous reports (Kuhara et al., 2008; Ohnishi et al., 2011). To monitor the temperature-evoked response of the AWC and AIY neurons, the yellow cameleon 3.6 was expressed in the wild-type N2 animals and *aho-3(nj15)* mutants. Each transgenic array containing *ceh-36p::yc3.60* or *ttx-3p::yc3.60* was transferred by outcrossing from N2 animals (Ohnishi et al., 2011; Kuhara et al., 2011) to *aho-3* mutants. We analyzed with well-fed and starved adult animals cultivated at 23˚C; animals were cultured and conditioned same as the population TTX assay, except only that animals were picked instead of washed when they were transferred from a NGM plate onto a starvation-conditioning plate. These animals were glued onto a 1.5% agar pad on glass, immersed in M9 buffer, and covered by cover glass. The agar pad and M9 buffer were kept at the initial imaging temperature (17˚C). The sample was then placed onto a peltier-based thermocontroller (Tokai Hit, Japan) on the stage of an Olympus BX61WI at the initial imaging temperature, and fluorescence was introduced into a Dual-View (Molecular devices, USA) optics systems. Cyan fluorescent protein (CFP; F480) and yellow fluorescent protein (YFP; F535) images were simultaneously captured by an EM-CCD camera C9100-13 ImagEM (Hamamatsu Photonics). Images were taken with a 500-ms exposure time or 150-ms exposure time with 2×2 binning. The temperature on the agar pad was monitored by a thermometer system, DCM-20 (Tokai Hit and Hamamatsu Photonics). For each

imaging experiment, fluorescence intensities of F535 and F480 were measured using MetaMorph imaging analysis system (Molecular Device). Since a computer regulates all the recorded images and the outcome of the analysis, any intention of a researcher should be excluded. Relative increases or decreases in the intracellular calcium concentration were measured as increases or decreases in the YFP/CFP fluorescence ratio of the cameleon protein (Ratio Change). The statistical analysis for ratio changes was performed by Steel-Dwass tests.

Supporting Figure Legends

Figure S1. Abnormal thermotactic plasticity of *aho-3* mutants on the 20°C-26°C thermal gradient.

(A-I) Thermotaxis of well-fed or starved wild-type and *aho-3(nj15)* mutant animals that were cultivated at 17°C (A-C), 20°C (D-F) or 23°C (G-I). $n \geq 4$ assays. Error bars represent SEM. In (A-B, D-E and G-H), statistical significance of values in each region was tested by unpaired *t*-test with the Dunn-Sidak correction for multiple comparisons; *, $p < 0.05$; **, $p < 0.01$. In (C, F and I), statistical significance of TTX indices was tested by unpaired *t*-test in comparisons of N2 animals vs *aho-3* mutants; *, $p < 0.05$; **, $p < 0.01$.

Figure S2. *aho-3* mutants show defects in the salt learning behavior and integration behavior.

(A) The salt chemotaxis learning assay (Tomioka *et al*., 2006) with some modification. The chemotaxis index was calculated by (A — B)/(total number of animals). See Supporting Experimental procedures for detail.

(B) Results of interaction assay of N2 animals, *aho-3(nj15)* mutants and transgenic animals cultivated at 20˚C. n ≥ 4 assays. Error bars represent SEM. Statistical significance of chemotaxis indices was tested by unpaired *t*-test in comparisons of N2 animals vs *aho-3* mutants and transgenic animals vs mutants; *, $p < 0.05$.

(C) Interaction assay for two opposite chemosensory stimuli Cu^{2+} ion and diacetyl (Ishihara et al., 2002). When these two stimuli were presented simultaneously, chemotaxis to attractant diacetyl was suppressed due to the repellent Cu^{2+} barrier. 1/100 diacetyl and 100mM $(CH_3COO)_2Cu$ were used. The index was defined as B/(A+B).

(D-F) Results of interaction assay of well-fed N2 animals, *hen-1(tm501)* mutants, *aho-3(nj15)* mutants and transgenic animals (only D) cultivated at 20˚C (D), 17˚C (E) or 25˚C (F). n ≥ 3 assays. Error bars represent SEM. Statistical significance of indices was tested by unpaired *t*-test in comparisons of N2 animals vs each mutants and transgenic animals vs *aho-3(nj15)* mutants; *, $p < 0.05$; **, $p < 0.01$.

(G-H) Results of avoidance from Cu^{2+} ion (G) and chemotaxis to diacetyl (H) of well-fed N2 animals and *aho-3(nj15)* mutants cultivated at 25˚C. n ≥ 3 assays. Error bars represent SEM. Unpaired *t*-test was used for comparisons of indices in each Cu^{2+} ion or odorant concentration; every comparison showed no significant difference ($p >$ 0.05).

Figure S3. Rescue experiment of *aho-3* mutants with K04G2.2 gene for the abnormal thermotactic plasticity.

(A-D) Thermotaxis of well-fed or starved wild-type N2 animals, *aho-3(nj15)* mutants and transgenic *aho-3(nj15)* mutants that were cultivated at 20°C (A-B) or 23°C (C-D). Transgenic mutants were carrying the PCR fragment containing the K04G2.2 gene region. n ≥ 4 assays. Error bars represent SEM. In (A and C), statistical significance of values in each region was tested by unpaired *t*-test with the Dunn-Sidak correction for multiple comparisons; *, $p < 0.05$; **, $p < 0.01$; colors of asterisks, red and green, represent comparisons of N2 animals vs mutants and transgenic animals vs mutants, respectively. In (B and D), statistical significance of TTX indices was tested by unpaired *t*-test in comparisons of N2 animals vs *aho-3* mutants and starved transgenic animals vs starved mutants; *, $p < 0.05$; **, $p < 0.01$.

Figure S4. Rescue experiments to identify the gene responsible for *aho-3* mutants.

(A) Four phenotypic categories based on the tracks of animals in the individual thermotaxis (TTX) assay with a 17°C–25°C thermal gradient (left); animals that moved to the cold region were classified as '17', animals that moved to the 20°C region were classified as '20', animals that moved to the warm region were classified as '25', and animals that moved to the cold and warm regions or moved like randomly were classified as '17/25' (Mohri et al., 2005).

(B-D) Results of individual TTX assays with well-fed or starved *aho-3(nj15)* mutants carrying cosmids or PCR fragments, cultivated at 25°C; #1, #2 and #3 indicate

independent transgenic lines; gfp indicates co-injection marker *ges-1p::NLS-GFP*. The cosmid K04G2 and PCR fragments, PCR1, PCR4-1 and PCR4-2, were containing the K04G2.2 gene region (see Figure 2A). Bars show the fraction of animals that moved to the 25°C region. Error bars represent SEM. n ≥ 3 assays. Tukey's test was used for multiple comparisons among values of starved animals; *, $p < 0.05$; **, $p < 0.01$; every comparison among lines #1, #2 and #3, carrying same transgene, showed no significant difference ($p > 0.05$). The line #2 carrying PCR 4-2 was used for experiments in Figure S2B, S2D and S3A-D.

Figure S5. Alignment of AHO-3 homologs and similar proteins.

Added to proteins in Figure 2C, secondary homolog in *S. mansoni* and similar proteins in *A. thaliana*, *O. sativa*, *S. pombe* and *D. discoideum* are shown. Identical residues are shaded in black, and similar residues are shaded in gray. Black bar represents conserved N-terminal cysteine cluster, and gray bar represents alpha/beta-hydrolase domain. Asterisks represent predicted catalytic residues. The *nj15* mutation results in Q to STOP at position 127. N-terminal sequences of protein in *D. discoideum* and C-terminal sequences of proteins in *S. mansoni*, *A. thaliana*, *O. sativa* and *D. discoideum* are omitted here.

Figure S6. AHO-3 novel protein is highly conserved among animal species.

Unrooted dendrogram of AHO-3 homologs and similar proteins in eleven animal species (black) and six non-animal species (blue). Gray highlights AHO-3 homolog

group, in which proteins share more than 70% amino acid sequence similarity with AHO-3 in the alpha/beta-hydrolase domain sequences and have conserved N-terminal cysteine cluster. Abbreviations used: Cel, *Caenorhabditis elegans*; Hsa, *Homo sapiens*; Dre, *Danio rerio*; Cin, *Ciona intestinalis*; Spu, *Strongylocentrotus purpuratus*; Dme, *Drosophila melanogaster*; Cte, *Capitella teleta*; Lgi, *Lottia gigantea*; Sma, *Schistosoma mansoni*; Nve, *Nematostella vectensis*; Tad, *Trichoplax adhaerens*; Sce, *Saccharomyces cerevisiae*; Spo, *Schizosaccharomyces pombe*; Ath, *Arabidopsis thaliana*; Osa, *Oryza sativa*; Cme, *Cyanidioschyzon merolae*; Ddi, *Dictyostelium discoideum*. Accession numbers are listed in Table S1.

Figure S7. A reporter gene expression under control of the *aho-3* promoter.

(A-B) The expression pattern of a reporter gene under control of the *aho-3* promoter. A general ER marker *cytochrome b5::yfp* (Rolls et al, 2002) was used as the reporter gene to show the expression in cell bodies clearer. The entire length of an adult animal (A) and a first stage larva (B). YFP fluorescence was observed in a subset of neurons, testes, hypodermis, pharyngeal muscle and intestine. Anterior is to the left. Z-stack confocal projections. Bars represent 30 μm.

(C-Q) The expression of the *aho-3p::cytochrome b5::yfp* in head neurons in adult animals (D, G, J, M and P). *odr-1p::cytochrome b5::cfp*, *gcy-8p::tagRFP*, *AIYp::tagRFP* and *tph-1p::NLS-tagRFP* were used as cell-specific markers (C, F, I, L and O). Merged images of upper panels are shown in (E, H, K, N and Q). In (G, H, J, K, M and N), strong YFP fluorescence in neighbor neurons is visible. Images shown in

(C-N) are high-magnification views of neurons shown in Figure 3A. Anterior is to the left. Single confocal sections. Bar represents 5 μm.

(R) The expression pattern of *aho-3p::gfp*. Merged image of a fluorescent micrograph with DIC micrograph. AFD, AWC, ADF and AIY neurons, that expressed GFP, were identified with their position and morphology. In this image, ADF and AIY neurons are out of focus. A head of a first stage larva is shown. Anterior is to the left. Bar represents 5 μm.

Figure S8. Cell-specific rescue experiments for *aho-3* mutants conditioned at 20°C.

(A-C) Thermotaxis of wild-type animals, *aho-3(nj15)* mutants and transgenic animals that were cultivated at 20°C with or without food. We used three promoters; *unc-14* promoter for pan-neuron, *odr-1* promoter for AWC and AWB and *ceh-36prom3* promoter for AWC. n ≥ 3 assays. Error bars represent SEM. Asterisks represent comparison of values in individual eight regions by unpaired *t*-test with the Dunn-Sidak correction for multiple comparisons of *aho-3* mutants vs transgenic animals; *, $p < 0.05$; **, $p < 0.01$. TTX indices of these data are shown in Figure 3D.

Figure S9. Locomotive ability of animals overexpressing AHO-3 in AWC.

Distributions of wild-type N2 animals, transgenic animals and *aho-3* mutants on TTX plates without temperature gradient. Well-fed animals cultivated at 20°C were placed at the center line of the plate and left for 60 min. Two lines of transgenic animals expressing AHO-3 in AWC and AWB under *odr-1* promoter were used. n ≥ 3 assays.

Error bars represent SEM. Unpaired *t*-test was used for comparisons of values in individual eight regions; every comparison showed no significant difference ($p > 0.05$).

Figure S10. The analysis with *aho-3;ceh-36* double mutants.

(A-B) Thermotaxis of double *aho-3(nj15);ceh-36(ky640)* and each single mutants cultivated at 20˚C with or without food. Results of N2 and *ceh-36(ky640)* mutants repeated from Figure 5. n ≥ 3 assays. Error bars represent SEM. In (A), asterisks represent comparison of values in individual eight regions by unpaired *t*-test with the Dunn-Sidak correction for multiple comparisons; *, $p < 0.05$; **, $p < 0.01$; colors of asterisks, red and blue, represent comparisons of double mutants to each single mutant, *aho-3(nj15)* and *ceh-36(ky640)*, respectively. Only when all "fraction" values in one dataset were "0.00", statistical analysis was not performed; in this case, we show a black cross representing double mutants. In (B), statistical significance of TTX indices was tested by unpaired *t*-test in comparisons of double mutants vs each single mutants; *, $p < 0.05$; **, $p < 0.01$. Error bars represent SEM.

Figure S11. Rescue experiment for the abnormal thermotactic plasticity of *odr-3* and *egl-4* mutants

(A-B) Thermotaxis of *odr-3(n1605)* mutants (A), *egl-4(n479)* mutants (B) and each transgenic animals cultivated at 23˚C with or without food. n ≥ 3 assays. Error bars represent SEM. Asterisks represent comparison of starved transgenic animals to starved mutant controls by Dunnett test; *, $p < 0.05$; **, $p < 0.01$.

Figure S12. Genetic relationship analysis among genes whose defect cause abnormal thermotactic plasticity; for 20˚C-cultivation

(A-H) Thermotaxis of double and single mutants cultivated at 20°C. Results of single mutants repeated from Figure 7A-B. n ≥ 3 assays. Error bars represent SEM. In (A-F), asterisks represent comparison of values in individual eight regions by unpaired *t*-test with the Dunn-Sidak correction for multiple comparisons; *, $p < 0.05$; **, $p < 0.01$; colors of asterisks, ocher, green, blue and purple, represent comparisons of double mutants to each single mutant, *odr-3(n1605)*, *gcy-28(tm2411)*, *egl-4(n479)* and *ins-1(nr2091)* mutants, respectively. Only when all "fraction" values in one dataset were "0.00", statistical analysis was not performed; in this case, we show a cross with colors, ocher, green and black, representing *odr-3(n1605)*, *gcy-28(tm2411)* and double mutants, respectively. In (G-H), TTX indices from data in (A-F) and Figure 7A-F are shown. Asterisks represent values different from single mutants by Dunnett test; *, $p < 0.05$; **, $p < 0.01$; ns, not significant ($p > 0.05$). Double crosses represent values different from N2 controls by Dunnett test; ‡, $p < 0.01$.

Figure S13. Genetic relationship analysis of *aho-3* with *odr-3*, *gcy-28* and *egl-4*; for 23˚C-cultivation.

(A-B) Thermotaxis of wild-type N2 and *aho-3(nj15)*, *odr-3(n1605)*, *gcy-28(tm2411)* and *egl-4(n479)* single-mutant animals cultivated at 23˚C with or without food.

(C-E) Thermotaxis of double and single mutants cultivated at 23˚C with or without food.

Results of single mutants repeated from (A-B).

In (A-E), asterisks represent comparison of values in individual eight regions by unpaired *t*-test with the Dunn-Sidak correction for multiple comparisons; *, $p < 0.05$; **, $p < 0.01$; colors of asterisks, ocher, green and blue, represent comparisons of N2 animals to (A-B) or double mutants to (C-E) each single mutant, *odr-3(n1605)*, *gcy-28(tm2411)* and *egl-4(n479)* mutants, respectively. Only when all "fraction" values in one dataset were "0.00", statistical analysis was not performed; in this case, we show a cross with colors, ocher, blue and black, representing *odr-3(n1605)*, *egl-4(n479)* and double mutants, respectively. n ≥ 3 assays. Error bars represent SEM.

(F-G) TTX indices from data in (A-E) are shown. Asterisks represent values different from *aho-3* mutants or N2 animals by Dunnett test; *, $p < 0.05$; **, $p < 0.01$; ns, not significant ($p > 0.05$). Unpaired *t*-test was used for comparisons between double mutants and *odr-3*, *gcy-28* or *egl-4* single mutants; †, $p < 0.05$; ‡, $p < 0.01$; ns, not significant ($p > 0.05$). Error bars represent SEM.

Figure S14. Genetic relationship analysis among *aho-3*, *odr-3* and *eat-16*

(A) Thermotaxis of wild-type N2 and *aho-3(nj15)*, *odr-3(n1605)* and *eat-16(nj8)* single-mutant animals cultivated at 20˚C with or without food.

(B-C) Thermotaxis of double and single mutants cultivated at 20˚C with or without food. Results of single mutants repeated from (A).

In (A-C), asterisks represent comparison of values in individual eight regions by unpaired *t*-test with the Dunn-Sidak correction for multiple comparisons; *, $p < 0.05$; **,

$p < 0.01$; colors of asterisks, red, ocher and blue, represent comparisons of N2 animals to (A) or double mutants to (B-C) each single mutant, *aho-3(nj15)*, *odr-3(n1605)* and *eat-16(nj8)* mutants, respectively. Only when all "fraction" values in one dataset were "0.00", statistical analysis was not performed; in this case, we show a cross with colors, red, ocher and black, representing *aho-3(nj15)*, *odr-3(n1605)* and double mutants, respectively. n ≥ 3 assays. Error bars represent SEM.

(D-E) TTX indices from data in (A-C). Asterisks represent values different from *eat-16(nj8)* mutants or N2 animals by Dunnett test; *, $p < 0.05$; **, $p < 0.01$; ns, not significant ($p > 0.05$). Unpaired *t*-test was used for comparisons between double and *aho-3* and *odr-3* single mutants; †, $p < 0.05$; ‡, $p < 0.01$; ns, not significant ($p > 0.05$). Error bars represent SEM.

Figure S15. Expressions and localizations of AHO-3 mutated in the predicted cataritic triad.

The fluorescence of AHO-3(wild-type)::EGFP (A), AHO-3(S191A, D256N and H285A)::EGFP (B) and AHO-3(S191A, D256N or H285A)::EGFP (C-E) expressed under control of the *unc-14* promoter in adult *aho-3(nj15)* mutants. Images are Z-stack confocal projection. Anterior is to the left. Bars represent 20 μm.

Figure S16. Subcellular localization assay of AHO-3.

Optical images of head neurons in adult *aho-3(nj15)* mutants (A-I) or wild-type animals (J-L) expressing marker-tagged proteins under control of the *aho-3* promoter.

(A-F) Images of AHO-3::CFP (A-B), Golgi marker mannosidase::YFP (C-D) and the overlay (E-F). Images shown in (A), (C) and (E) are z-stack confocal projection, and a rough outline of head of animal is shown (thick dashed line). Images shown in (B), (D) and (F) are single confocal section, that are high-magnification views of a cell body shown in (A), (C) and (E). Solid arrowheads point to the AHO-3::CFP localization to sensory endings. Bars represent 10 μm.

(G-I) Images of AHO-3::CFP (G), Golgi marker MIG-23::GFP (H) and the overlay (I). Images are single confocal section. Bars represent 5 μm.

(J-L) Images of AHO-3::dsRed-monomer (J), general ER marker Cytochrome-b5::YFP (K) and the overlay (L). Images are z-stack confocal projection. Bars represent 5 μm.

Anterior is to the left for all panels.

Figure S17. Expressions and localizations of AHO-3 mutated in the N-terminal cysteines.

The fluorescence of AHO-3(wild-type)::EGFP (A), AHO-3(C34S, C35S, C38S and C39S)::EGFP (B) and AHO-3(C34S, C35S, C38S or C39S)::EGFP (C-F) expressed under control of the *unc-14* promoter in adult *aho-3(nj15)* mutants. Images are Z-stack confocal projection. Anterior is to the left. Bars represent 20 μm.

Figure S18. *in vivo* calcium imaging of AWC and AIY according to temperature change in *aho-3* mutants.

(A-B, D-E) Calcium imaging of the AWC sensory neuron (A-B) and AIY interneuron

(D-E) in animals, cultivated at 23˚C under well-fed condition (A, D) or starved condition (B, E). The average percentage changes in normalized YFP/CFP ratio (R/R0) were represented black (N2 animals) and red (*aho-3(nj15)* mutants) traces. n = 25–28 (A-B), n = 11–14 (D-E). Gray and pink shading indicates SEM. Temperature change is shown at the each bottom. 0 sec is the time starting temperature change from 17˚C to 23˚C.

(C, F) The average of Ratio changes to temperature stimuli at 95 sec regarding the results shown in (A-B) and (D-E). All comparisons among well-fed and starved animals showed no significant difference ($p > 0.05$) by Steel-Dwass tests for multiple comparison.

(G) Thermotaxis of N2 animals, *aho-3(nj15)* mutants and each transgenic animals carrying cameleon (*yc3.60*). Animals were cultivated at 23˚C with or without food. n = 3 assays. Error bars represent SEM. Tukey's test was used for multiple comparisons among TTX indices of starved animals or among that of well-fed animals; *, $p < 0.05$; **, $p < 0.01$; ns, not significant ($p > 0.05$). TTX indices of transgenic animals were not significantly different from that of control animals in each condition, except starved N2; Ex[*AIYp::yc3.60*, *gcy-8p::yc3.60*].

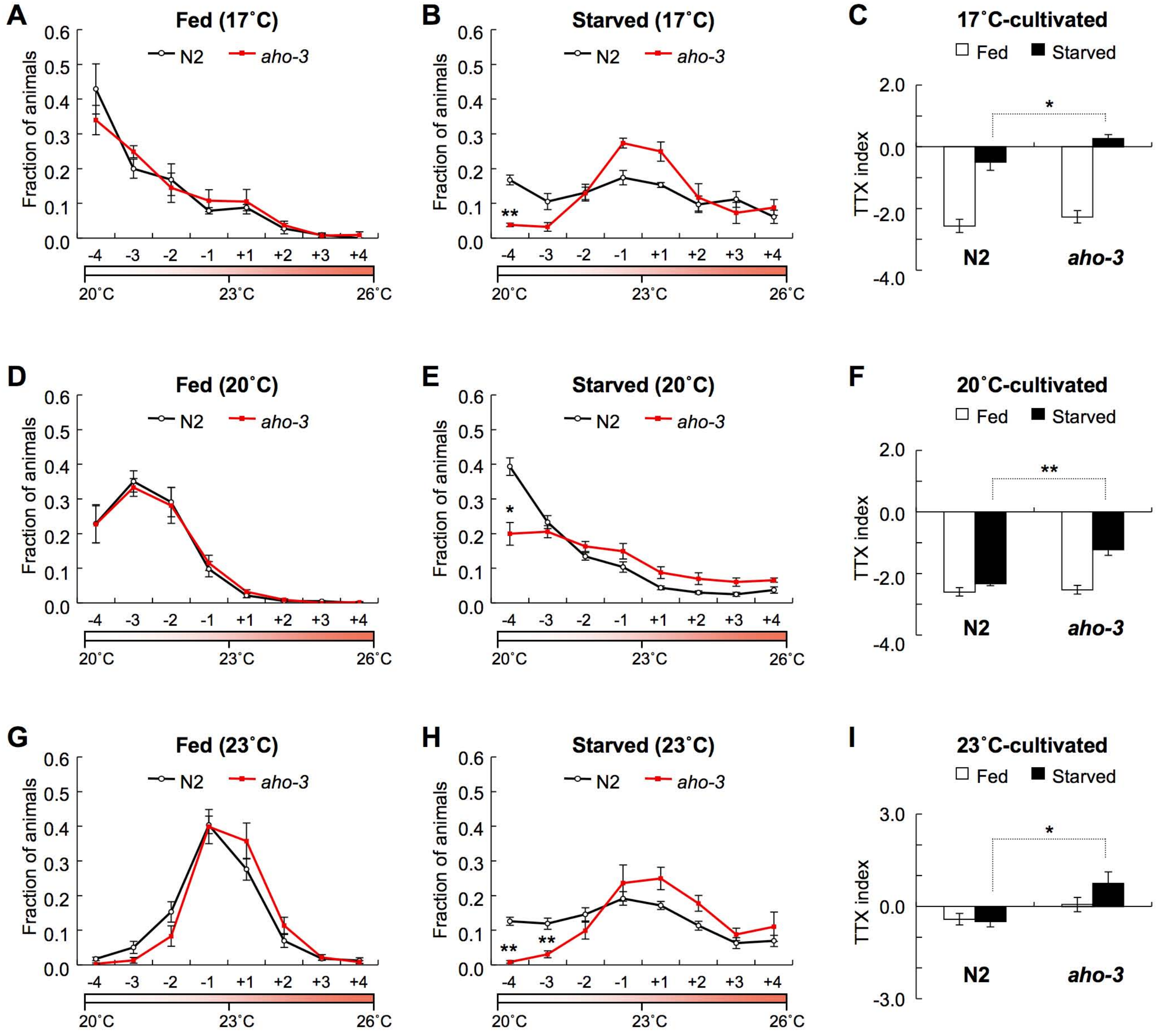
A
Fed (17˚C)
B
Starved (17˚C)
C
17˚C-cultivated
D
Fed (20˚C)
E
Starved (20˚C)
F
20˚C-cultivated
G
Fed (23˚C)
H
Starved (23˚C)
I
23˚C-cultivated
N2
aho-3
Fed
Starved
Fraction of animals
TTX index
-4 -3 -2 -1 +1 +2 +3 +4
20˚C
23˚C
26˚C

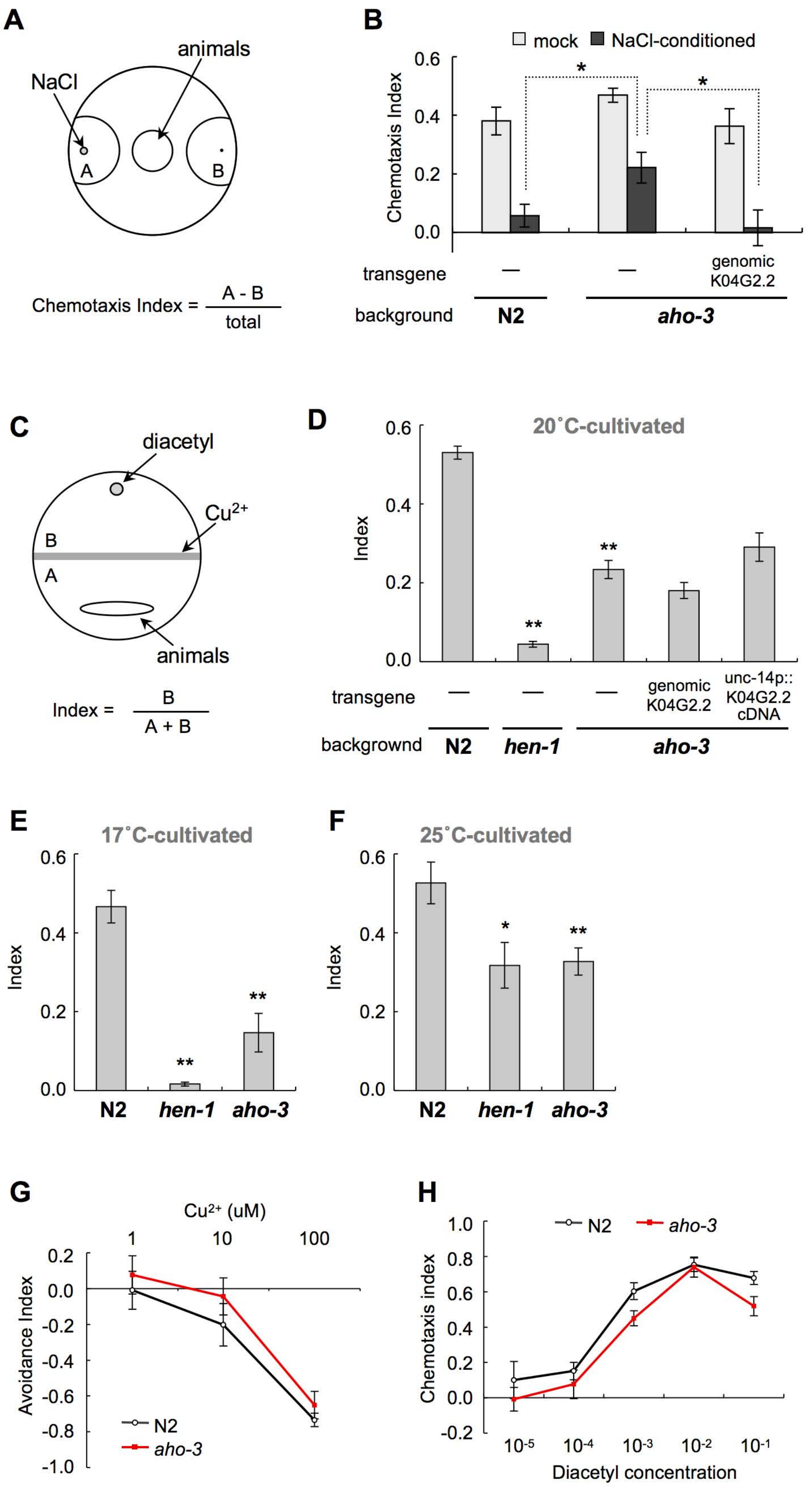
A
animals
NaCl
A
B
Chemotaxis Index = A - B / total
B
mock
NaCl-conditioned
0.6
0.4
0.2
0.0
Chemotaxis Index
*
*
transgene
—
—
genomic K04G2.2
background
N2
aho-3
C
diacetyl
Cu2+
B
A
animals
Index = B / A + B
D
20˚C-cultivated
0.6
0.4
0.2
0.0
Index
**
**
transgene
—
—
—
genomic K04G2.2
unc-14p:: K04G2.2 cDNA
backgrownd
N2
hen-1
aho-3
E
17˚C-cultivated
0.6
0.4
0.2
0.0
Index
**
**
N2
hen-1
aho-3
F
25˚C-cultivated
0.6
0.4
0.2
0.0
Index
*
**
N2
hen-1
aho-3
G
Cu2+ (uM)
1
10
100
0.2
0.0
-0.2
-0.4
-0.6
-0.8
-1.0
Avoidance Index
N2
aho-3
H
1.0
0.8
0.6
0.4
0.2
0.0
-0.2
Chemotaxis index
N2
aho-3
10-5
10-4
10-3
10-2
10-1
Diacetyl concentration

Nishio *et al.*, Figure S3

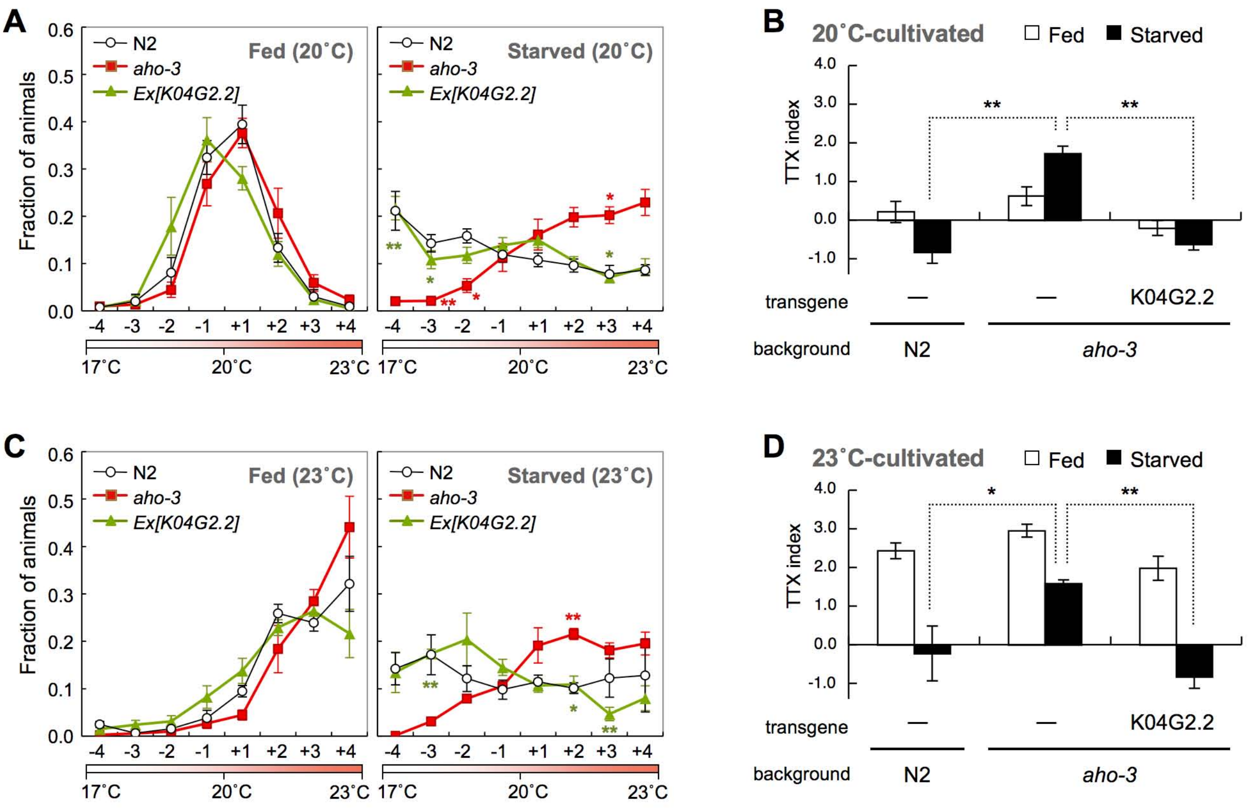

Nishio *et al.*, Figure S4

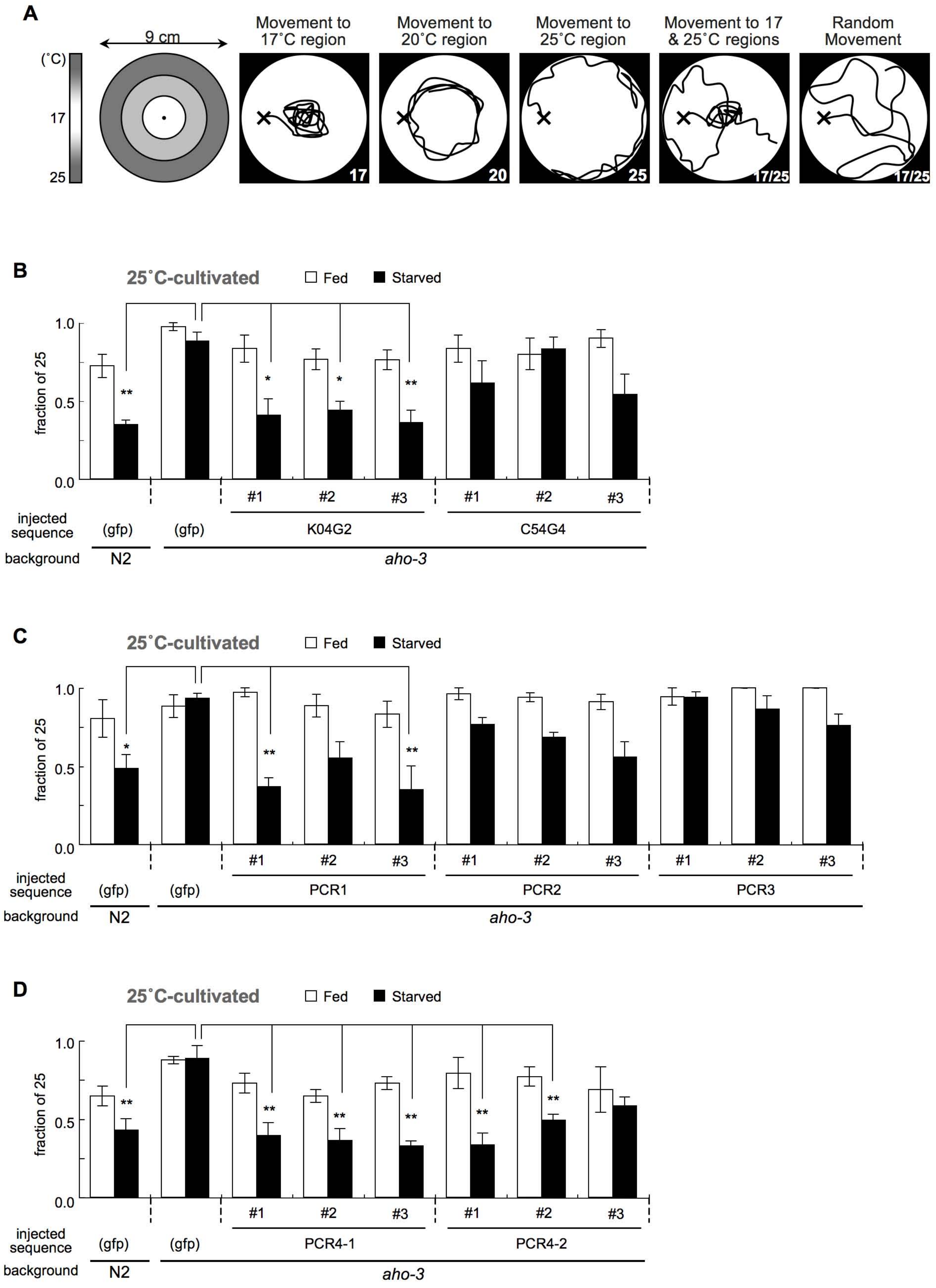

Nishio *et al.*, Figure S5

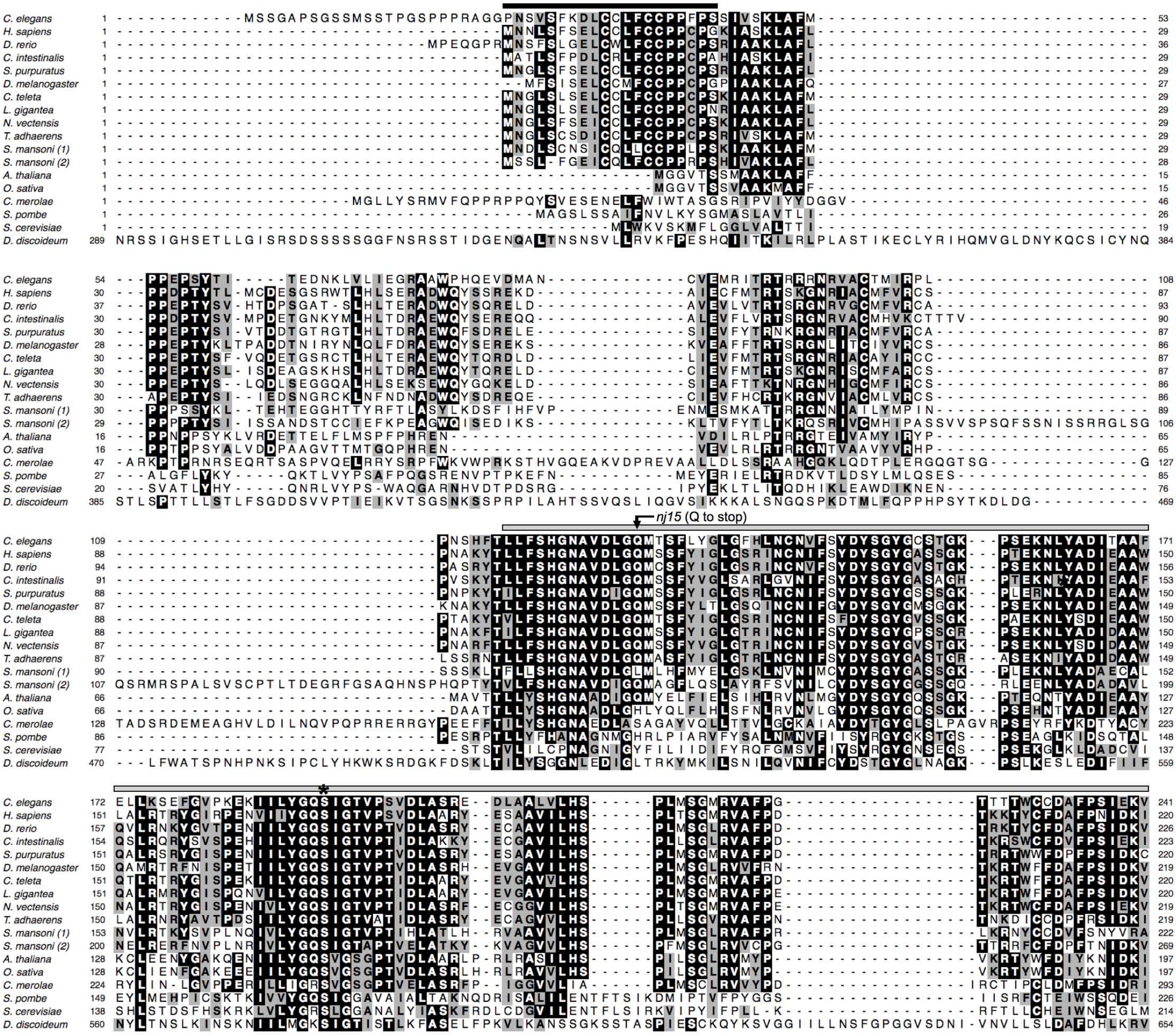

Nishio *et al.*, Figure S6

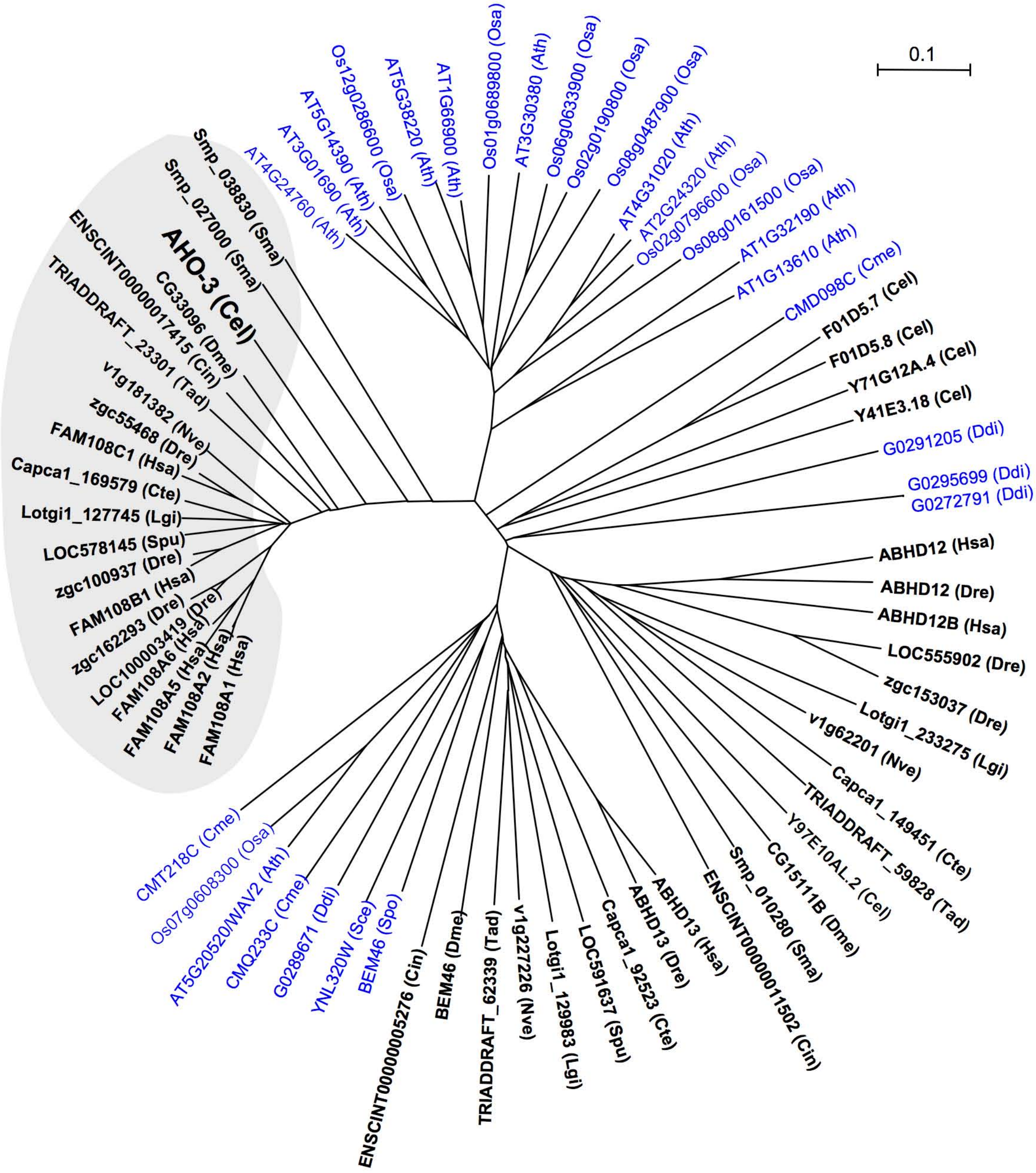

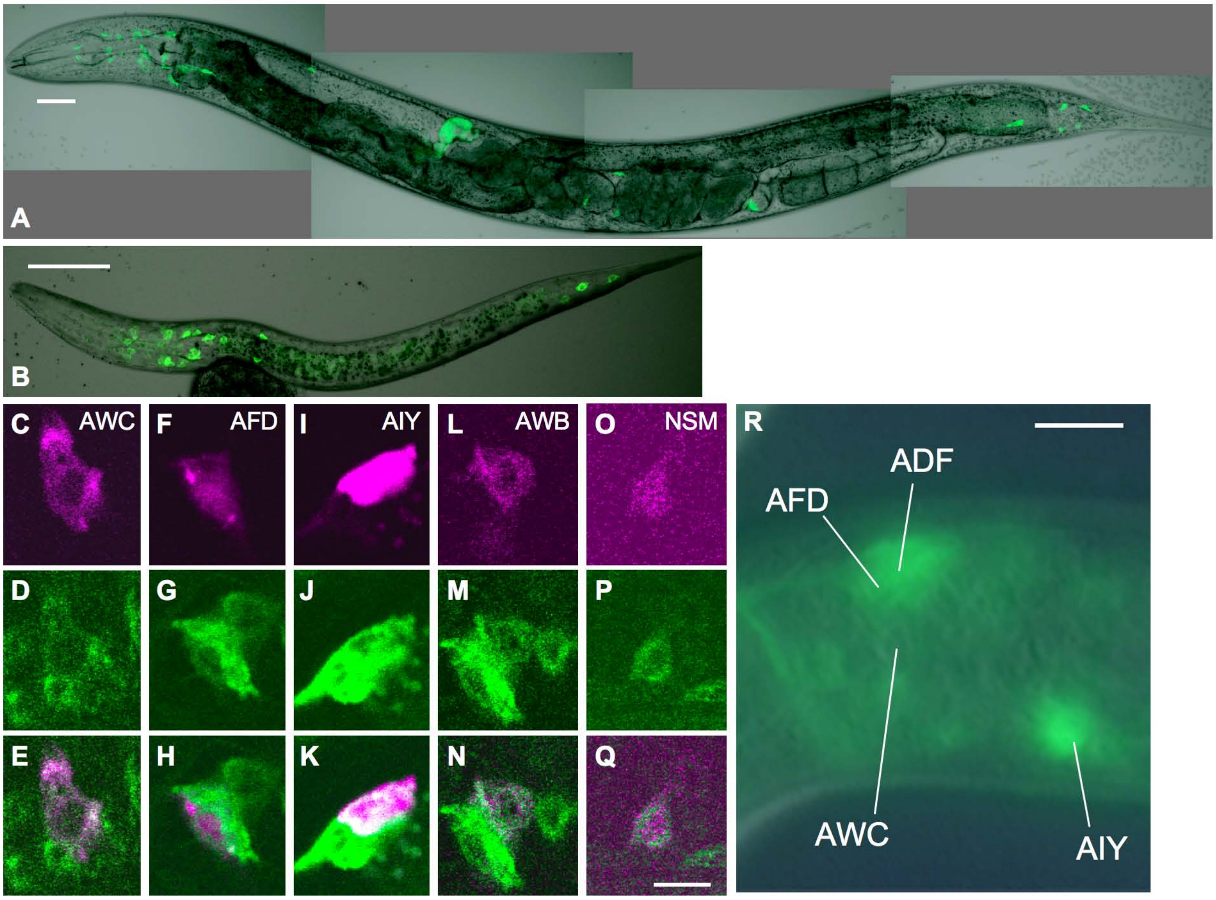
A
B
C
AWC
F
AFD
I
AIY
L
AWB
O
NSM
D
G
J
M
P
E
H
K
N
Q
R
ADF
AFD
AWC
AIY

Nishio *et al.*, Figure S8

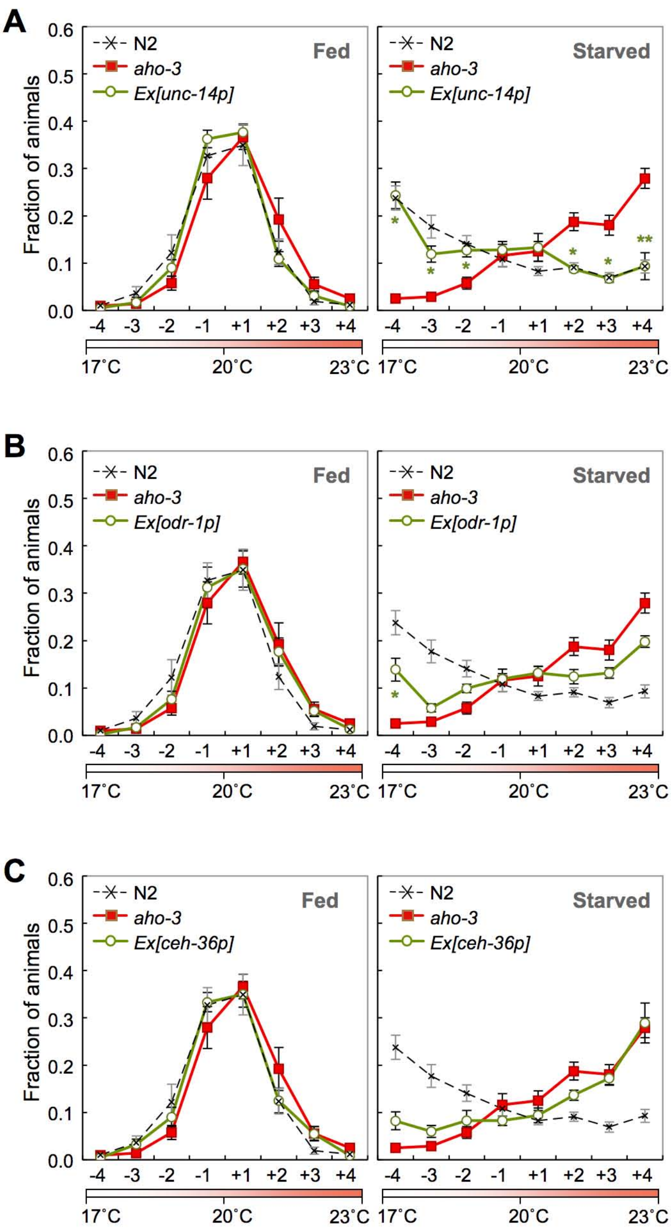

Nishio *et al.*, Figure S9

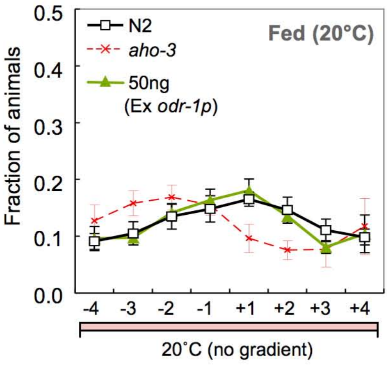

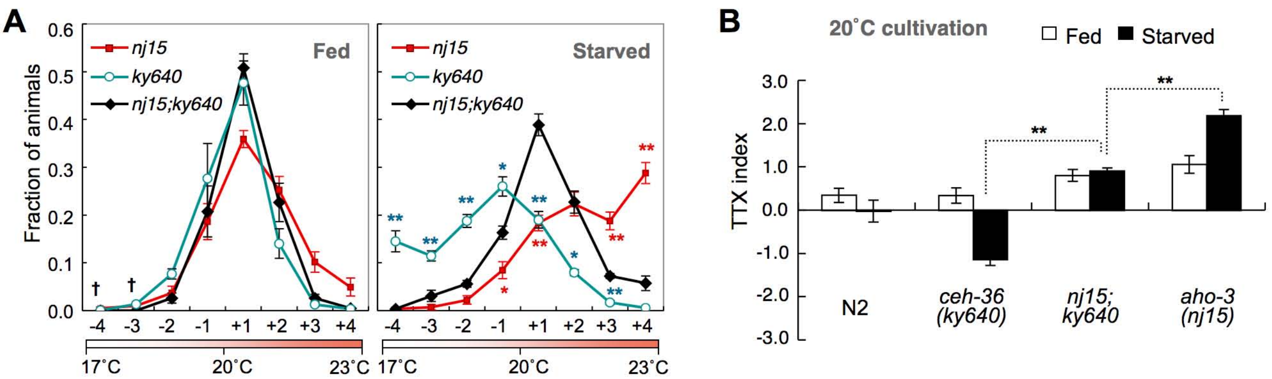
A
Fraction of animals
nj15
ky640
nj15;ky640
Fed
Starved
0.6
0.5
0.4
0.3
0.2
0.1
0.0
-4
-3
-2
-1
+1
+2
+3
+4
17˚C
20˚C
23˚C
B
20˚C cultivation
Fed
Starved
TTX index
3.0
2.0
1.0
0.0
-1.0
-2.0
-3.0
N2
ceh-36 (ky640)
nj15; ky640
aho-3 (nj15)

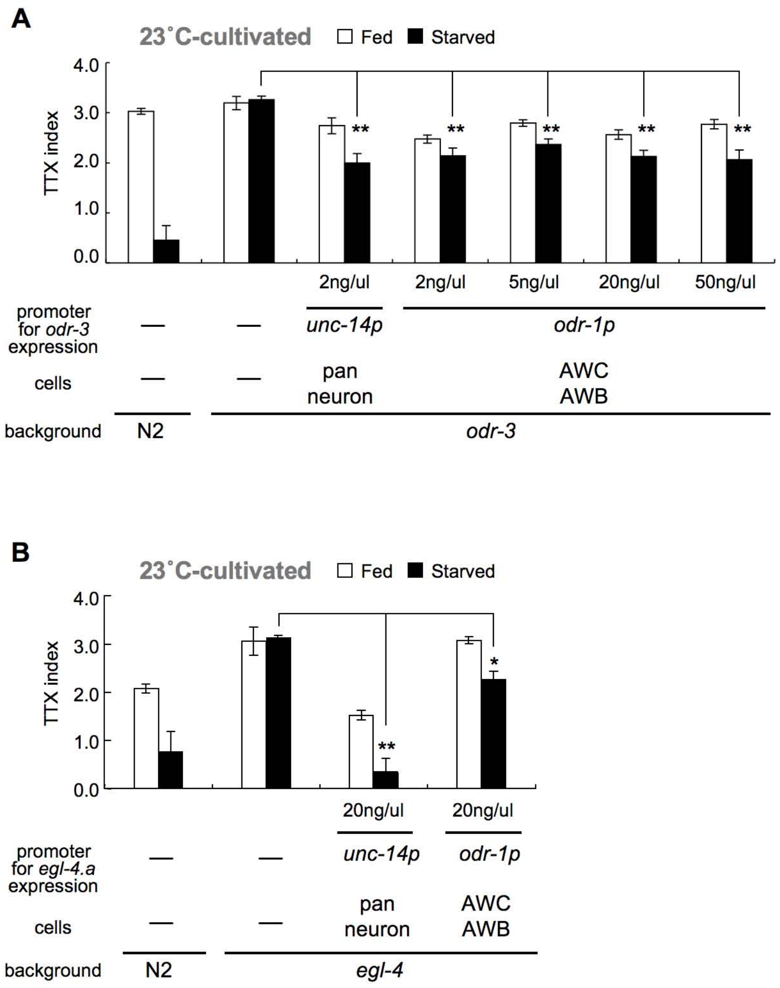
A
23˚C-cultivated
Fed
Starved
TTX index
4.0
3.0
2.0
1.0
0.0
**
2ng/ul
2ng/ul
5ng/ul
20ng/ul
50ng/ul
promoter for odr-3 expression
unc-14p
odr-1p
cells
pan neuron
AWC AWB
background
N2
odr-3
B
23˚C-cultivated
Fed
Starved
TTX index
*
20ng/ul
20ng/ul
promoter for egl-4.a expression
unc-14p
odr-1p
cells
pan neuron
AWC AWB
background
N2
egl-4

Nishio *et al.*, Figure S12

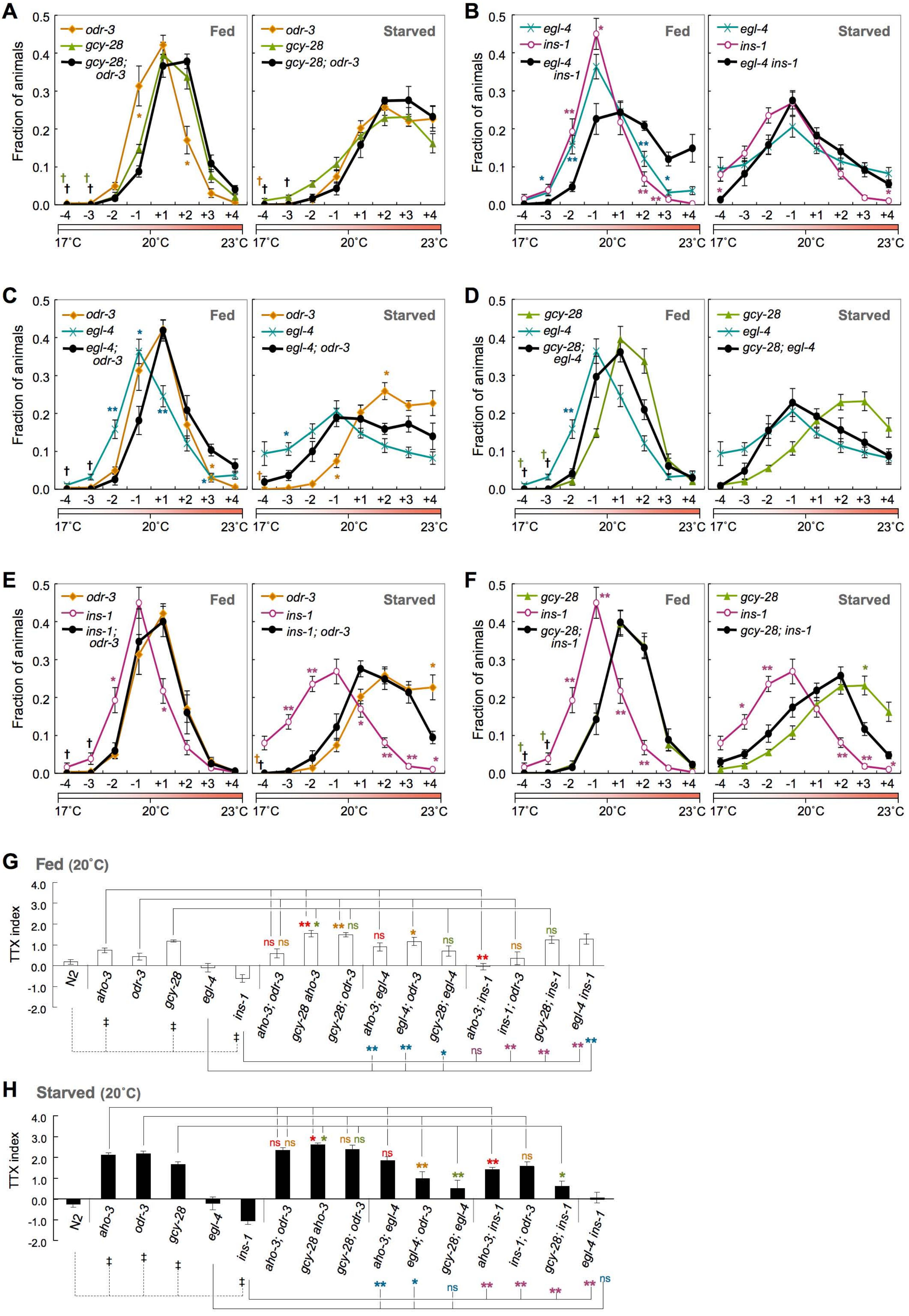

Nishio *et al.*, Figure S13

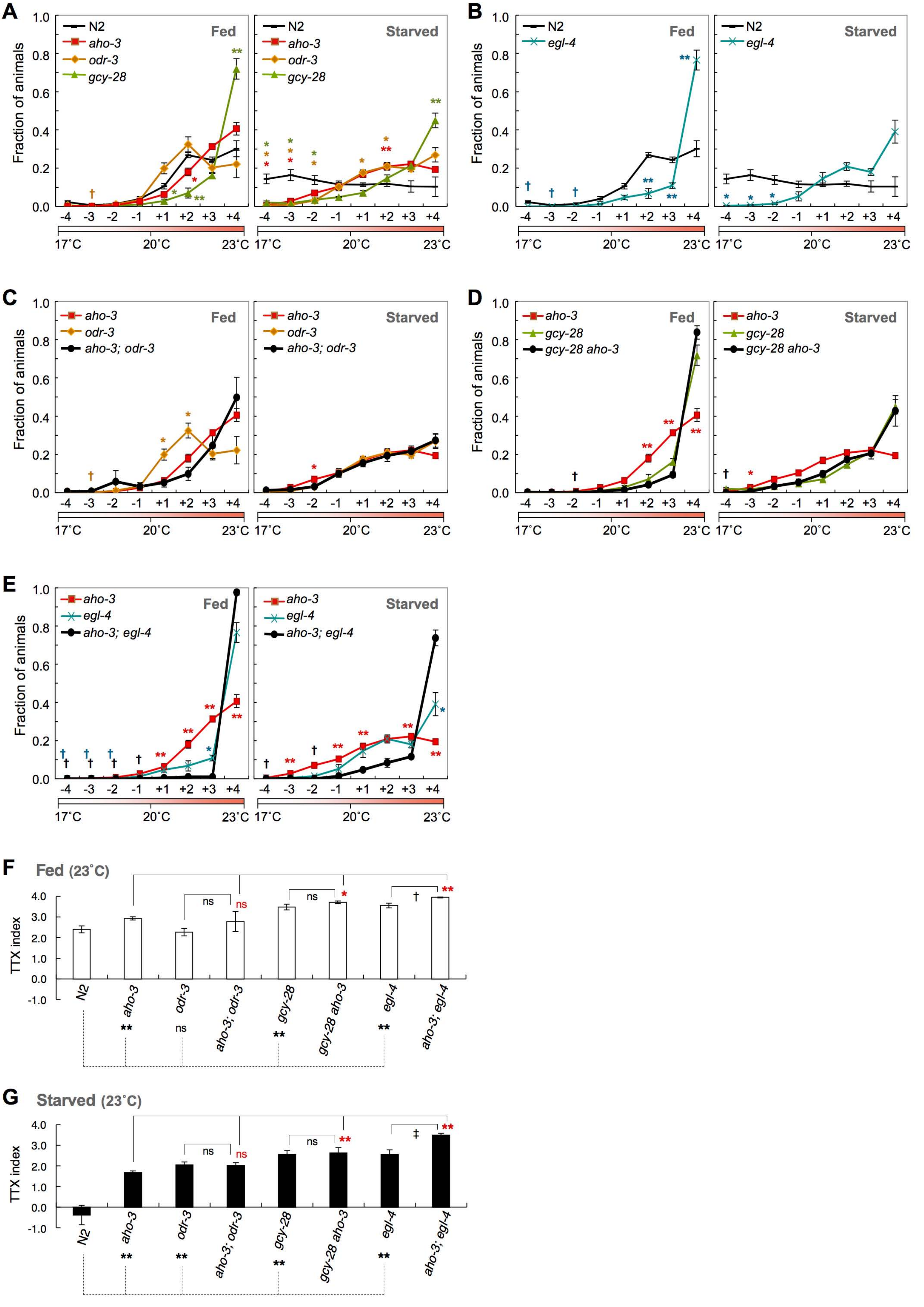

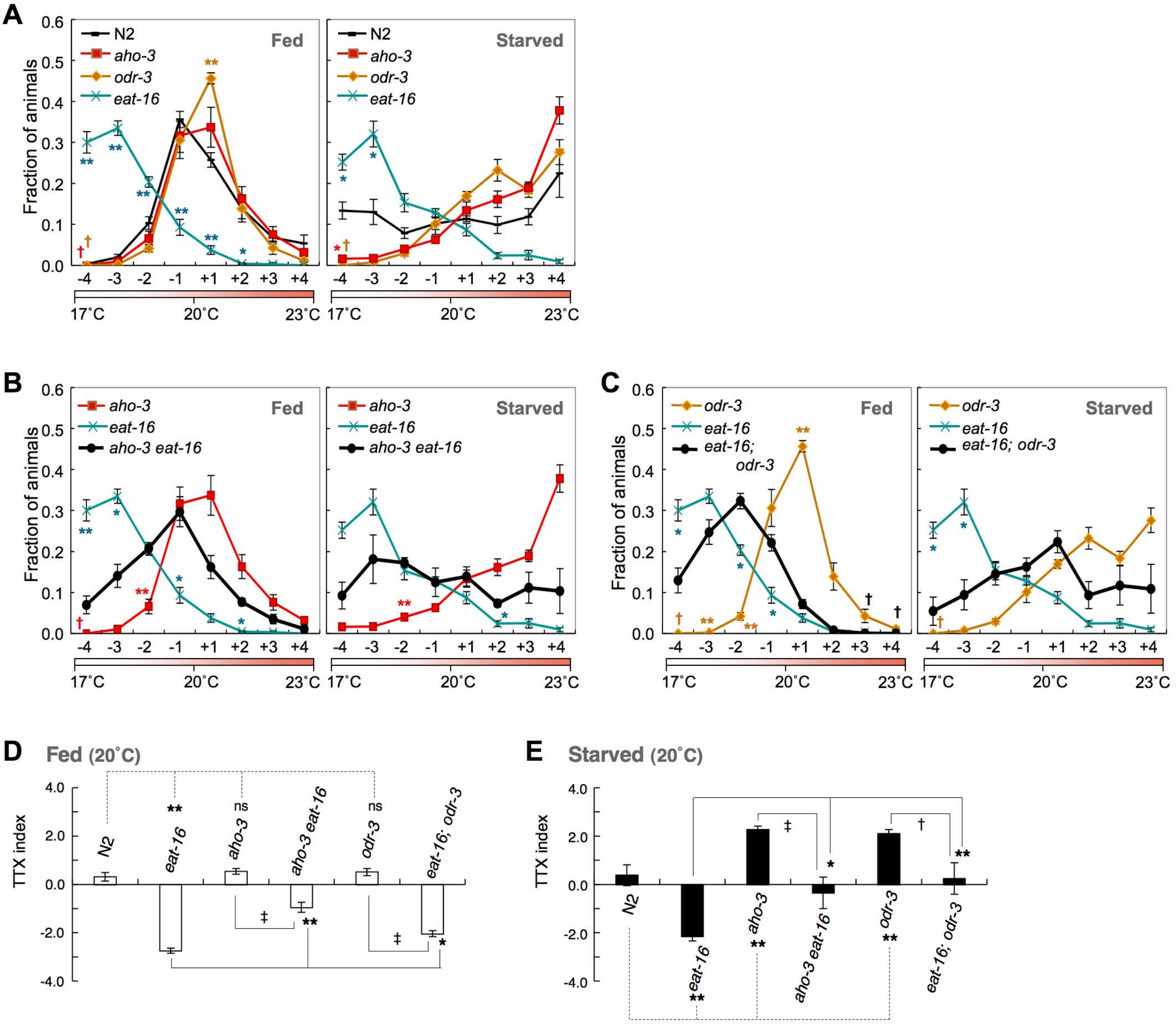
A
B
C
D
E
Fed
Starved
N2
aho-3
odr-3
eat-16
aho-3 eat-16
eat-16; odr-3
Fraction of animals
17˚C
20˚C
23˚C
Fed (20˚C)
Starved (20˚C)
TTX index
ns

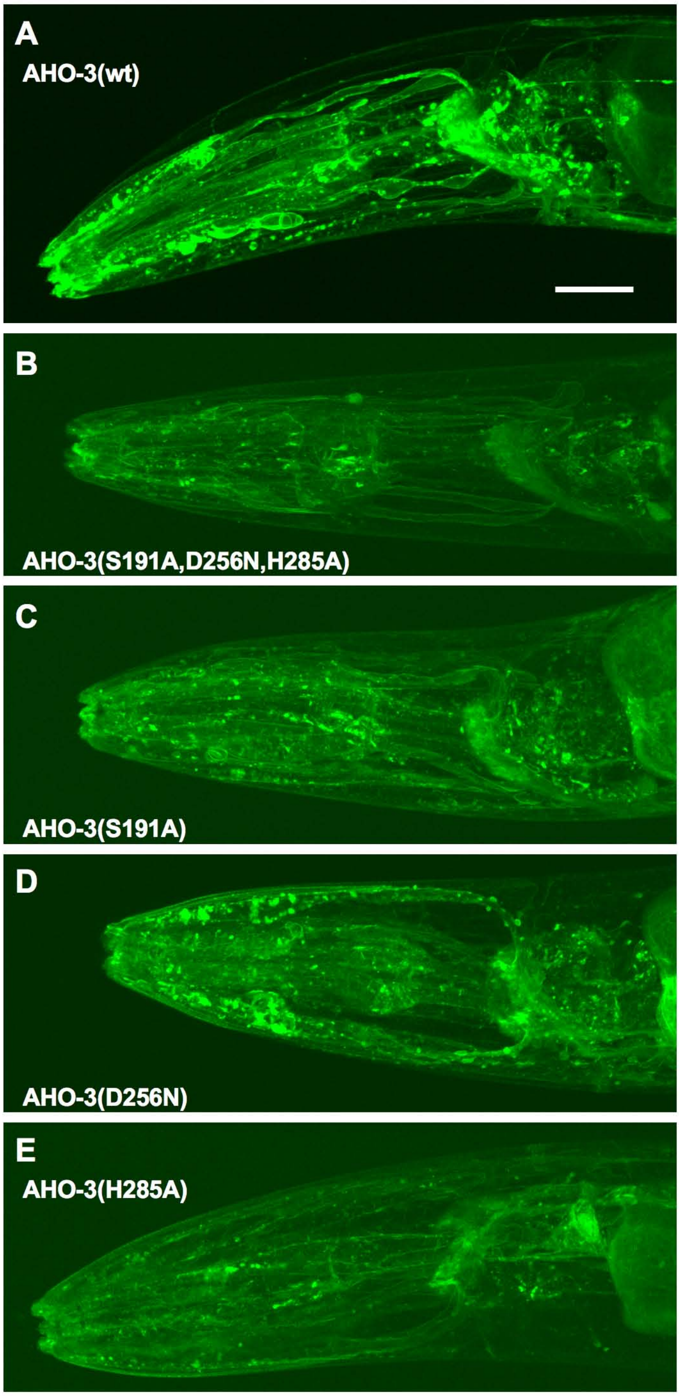
A
AHO-3(wt)
B
AHO-3(S191A,D256N,H285A)
C
AHO-3(S191A)
D
AHO-3(D256N)
E
AHO-3(H285A)

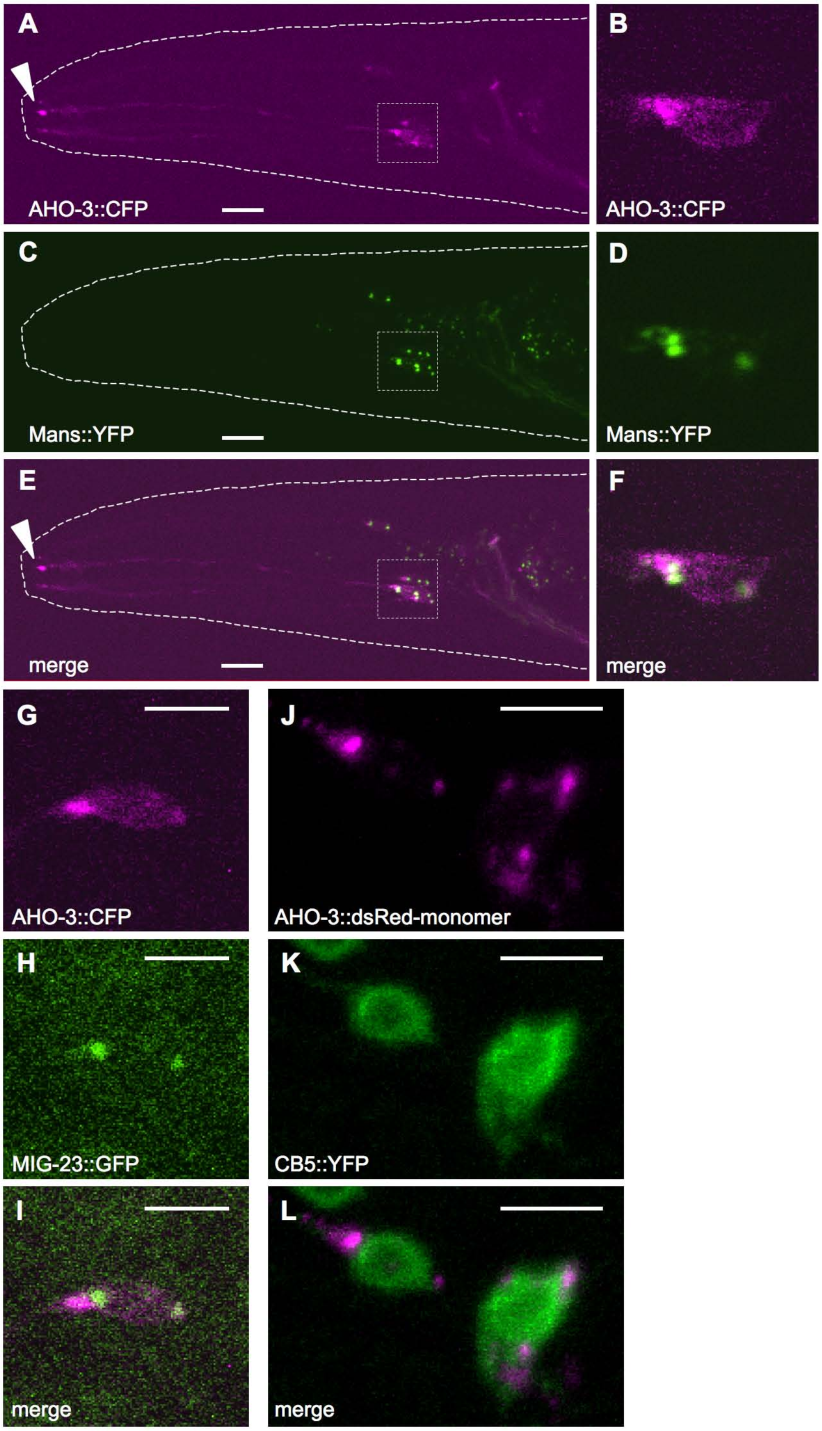
A
AHO-3::CFP
B
AHO-3::CFP
C
Mans::YFP
D
Mans::YFP
E
merge
F
merge
G
AHO-3::CFP
J
AHO-3::dsRed-monomer
H
MIG-23::GFP
K
CB5::YFP
I
merge
L
merge

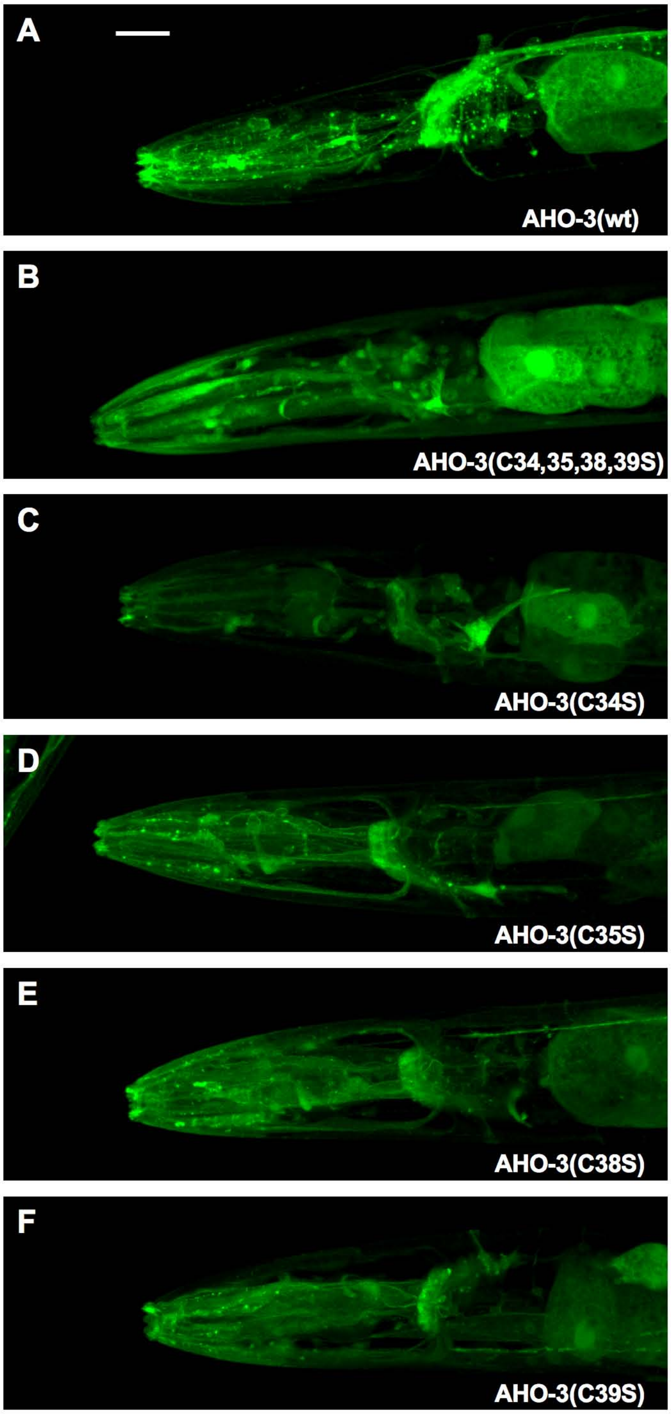
A
AHO-3(wt)
B
AHO-3(C34,35,38,39S)
C
AHO-3(C34S)
D
AHO-3(C35S)
E
AHO-3(C38S)
F
AHO-3(C39S)

Nishio *et al.*, Figure S18

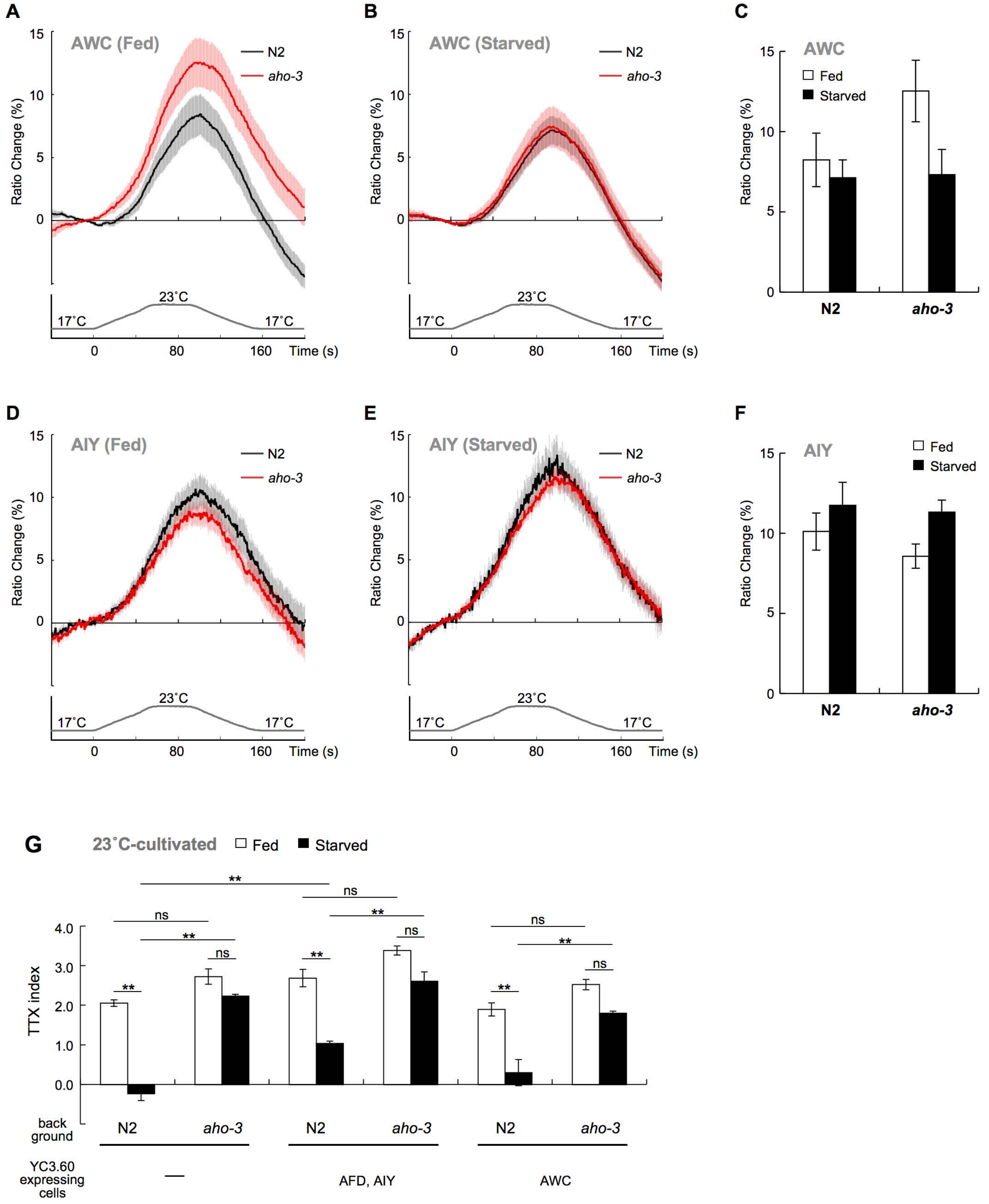

Table S1. AHO-3 homologs and similar proteins

Accession Number	Gene or Product Name	Biological Species
NP_492210.2	K04G2.2/ *aho-3*	*Caenorhabditis elegans*
NP_001022066.1	F01D5.7	
NP_496938.1	F01D5.8	
NP_001023462.1	Y41E3.18	
NP_490914.2	Y71G12A.4	
NP_505054.1	Y97E10AL.2	
NP_112490.3	FAM108A1	*Homo sapiens*
CAI23639.1	FAM108A2	
XP_001721981.1	FAM108A5	
A6NEC5.1	FAM108A6	
NP_001020951.1	FAM108B1	
NP_067037.1	FAM108C1	
NP_116248.2	ABHD13	
NP_056415.1	ABHD12	
NP_853511.2	ABHD12B	
XP_001342996.1	LOC100003419	*Danio rerio*
NP_956451.1	zgc:55468	
CAM56648.1	zgc:100937	
NP_001082792.1	zgc:162293	
NP_001032774.1	abhd13	
NP_001070065.1	abhd12	
XP_683654.2	LOC555902	
NP_001038808.1	zgc:153037	
ENSCINT00000017415.2	CIPRO316.4.1	*Ciona intestinalis*
ENSCINT00000005276.2	CIPRO821.7.1	
ENSCINT00000011502.2	CIPRO37.53.1	
XP_001192514.1	LOC578145	*Strongylocentrotus purpuratus*
XP_001181066.1	LOC591637	
NP_788737.1	CG33096	*Drosophila melanogaster*
NP_477372.1	Bem46	
NP_725856.1	CG15111B	
jgi|Capca1|169579	estExt_Genewise1Plus.C_610060	*Capitella teleta*
jgi|Capca1|92523	e_gw1.67.105.1	
jgi|Capca1|149451	estExt_Genewise1.C_380129	
jgi|Lotgi1|127745	e_gw1.58.247.1	*Lottia gigantea*
jgi|Lotgi1|129983	e_gw1.67.286.1	
jgi|Lotgi1|233275	estExt_fgenesh2_pg.C_sca_370064	
XP_002574140.1	Smp_027000	*Schistosoma mansoni*
XP_002575250.1	Smp_038830	
XP_002572492.1	Smp_010280	
XP_001637806.1	v1g181382	*Nematostella vectensis*
XP_001628709.1	v1g227226	
XP_001640760.1	v1g62201	
XP_002111042.1	TRIADDRAFT_23301	*Trichoplax adhaerens*
XP_002118287.1	TRIADDRAFT_62339	
XP_002115921.1	TRIADDRAFT_59828	
NP_014079.1	YNL320W	*Saccharomyces cerevisiae*
NP_595609.1	bem46	*Schizosaccharomyces pombe*
NP_194207.1	AT4G24760	*Arabidopsis thaliana*
NP_001154655.1	AT3G30380	
NP_186818.1	AT3G01690	
NP_196943.1	AT5G14390	
NP_194831.3	AT4G31020	
NP_180009.2	AT2G24320	
NP_001031978.1	AT5G38220	
NP_176862.2	AT1G66900	
NP_001077642.1	AT1G32190	
NP_001031042.1	AT1G13610	
NP_568395.1	AT5G20520/ WAV2	
NP_001066588.1	Os12g0286600	*Oryza sativa japonica*
NP_001058133.1	Os06g0633900	
NP_001043924.2	Os01g0689800	
NP_001046153.1	Os02g0190800	
NP_001048390.1	Os02g0796600	
NP_001062104.1	Os08g0487900	
NP_001061050.1	Os08g0161500	
NP_001060241.1	Os07g0608300	
CMD098C	CMD098C	*Cyanidioschyzon merolae*
CMQ233C	CMQ233C	
CMT218C	CMT218C	
XP_635345.1	G0291205	*Dictyostelium discoideum*
XP_636045.1	G0289671	
XP_644406.2	G0295699	
XP_645007.1	G0272791	

Table S2. Expression patterns driven by each promoter

Promoter	Expression pattern	Reference
unc-14p	almost all neurons	(Ogura et al, 1997)
osm-6p	ADF, AFD, AWA, AWB, AWC, ASE, ASG, ASH, ASI, ASJ, PHA, PHB, IL2… (sensory neurons)	(Collet et al, 1998; Kodama et al, 2006)
ncs-1p	ADF, AFD, AWA, AWB, AWC, AIY, ASE, ASG, AVK, BAG, PHA, PHB, RMG, pm1	(Gomez et al, 2001)
glr-1p	AIB, AVA, AVB, AVD, AVE, AVG, AVJ, DVC, PVC, PVQ, RIG, RIM, RIS, RMD, RME, SMD, URY	(Hart et al, 1995; Maricq et al, 1995)
glr-2p	AIA, AIB, AVA, AVD, AVE, AVG, PVC, RIA, RIG, RMD, pm1, RIR(?)	(Brockie et al, 2001)
odr-1p	AWB, **AWC**	(L'Etoile & Bargmann, 2000)
ceh-36p	AWC	(Etchberger et al, 2007)
gcy-8p	AFD	(Inada et al, 2006)
AIYp	AIY	(Kodama et al, 2006; Kuhara & Mori, 2006)
tph-1p	ADF, HSN, NSM	(Sze et al, 2000)
srh-142p	ADF	(Chang & Bargmann, 2008; Sagasti et al, 1999)
glr-3p	RIA	(Brockie et al, 2001)
str-1p	AWB	(Troemel et al, 1997)

Table S3. Subcellular localization analysis of AHO-3 with fed and starved animals

subcellular location	Fed / Starved	Category AHO-3 fluorescence strong	weak	invisible	Total *n*
sensory ending	Fed	21	18	6	45
	Starved	20	18	5	43
cell body dots	Fed	32	10	3	45
	Starved	22	18	3	43
cell body diffuse	Fed	10	24	11	45
	Starved	8	30	5	43

Fluorescence levels of subcellular locations were observed in fed and starved adults carrying *aho-3p::aho-3cDNA::egfp*. Animals were cultivated at 23°C. We categorized fluorescence intensity in sensory endings and cell bodies to strong, weak, and invisible. Statistical analysis by a chi-square test using a 2 x 3 contingency table was performed to compare between fed animals and starved animals. Each comparison shows no significant difference.
